# A taxonomic guide to the brittle-stars (Echinodermata, Ophiuroidea) from the State of Paraíba continental shelf, Northeastern Brazil

**DOI:** 10.3897/zookeys.307.4673

**Published:** 2013-06-10

**Authors:** Anne I. Gondim, Carmen Alonso, Thelma L. P. Dias, Cynthia L. C. Manso, Martin L. Christoffersen

**Affiliations:** 1 Universidade Federal da Paraíba, Programa de Pós-Graduação em Ciências Biológicas (Zoologia), Laboratório de Invertebrados Paulo Young (LIPY), João Pessoa, PB.CEP. 58059-900, Brasil; 2 Universidade Estadual da Paraíba, Laboratório de Biologia Marinha (LabMar), Departamento de Biologia, Campus I, Rua Baraúnas, 351, Bairro Universitário, CEP 58429-500, Campina Grande, PB, Brasil; 3 Universidade Federal de Sergipe, Departamento de Biociências, Laboratório de Invertebrados Marinhos (LABIMAR). Av. Vereador Olimpio Grande S/nº, 49.500-000, Itabaiana, SE, Brasil

**Keywords:** Echinoderms, Ophiurida, checklist, Brazilian coast, distribution

## Abstract

We provide the first annotated checklist of ophiuroids from the continental shelf of the State of Paraíba, northeastern Brazil. Identification keys and taxonomic diagnoses for 23 species, belonging to 14 genera and 8 families, are provided. The material is deposited in the Invertebrate Collection Paulo Young, at the Federal University of Paraíba. *Ophiopsila hartmeyeri* represents the first record for the northeastern region of Brazil, while *Ophiolepis impressa*, *Ophiolepis paucispina*, *Amphiura stimpsoni*, *Amphiodia riisei*, *Ophiactis quinqueradia*, *Ophiocoma wendtii* and *Ophionereis olivaceae* are new records for the State of Paraíba. The number of species known for the state was increased from 16 to 23, representing approximately 17% of the species known for Brazil and 54% of the species known for northeastern Brazil. The recorded fauna has a large geographical and bathymetrical distribution.

## Introduction

The class Ophiuroidea includes the most agile and diverse animals within the phylum Echinodermata (Hyman 1955). Their representatives live associated with diverse substrates in all seas, oceans and depths (Borges and Amaral 2005). Although ophiurans are common and conspicuous animals, the scientific effort to describe their diversity has varied over the centuries, resulting in patchy knowledge (Stöhr et al. 2012).

The study of ophiuroids in Brazil started with Lyman (1875), who described the results of the Hassler Expedition, off Bahia and Rio de Janeiro (Borges and Amaral 2007). Occasional records of the occurrence of some species had already been made ​​prior to the study of Lyman (e.g. Marcgrave 1648, Verrill 1868). However, knowledge on the diversity of Ophiuroidea remains scarce in Brazil, with 134 recorded species (Barboza and Borges 2012, Gondim et al. 2012). The north and northeastern coasts are poorly known, the State of Paraíba remaining one of the least studied regions in northeast Brazil. Existing knowledge to the Paraíba coast is limited to occasional citations in Rathbun (1879), Lyman (1882), H.L. Clark (1915), Tommasi (1970), Thomas (1973), Albuquerque (1986), Albuquerque and Guille (1991), Young (1986), and Gondim et al. (2008, 2010, 2011).

**Figure 1.**
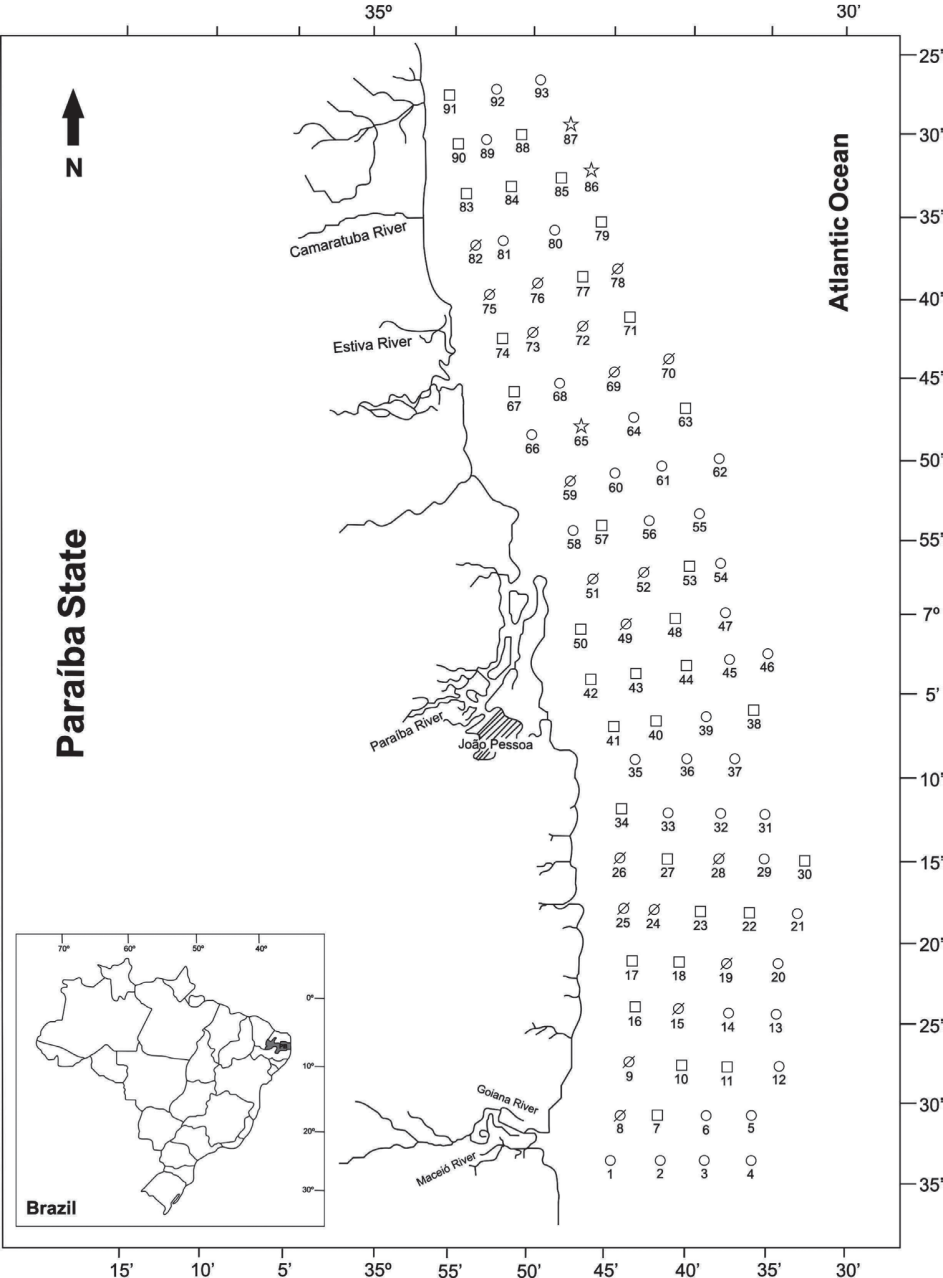
Collecting stations of Project Algae along the coast of the State of Paraíba, northeastern Brazil, with indication of the abundance of specimens in each site. Ø = absent in the collection point, □ = occurrence of 1 to 10 specimens, ○ = occurrence of 11 to 50 specimens, and ☆ = occurrence of more than 50 specimens.

The coast of the State of Paraíba (Fig. 1) has 138 km and its continental shelf is narrow, shallow and relatively plane, with a mean width of 30 km, becoming broader in a north-south direction (Feitosa et al. 2005). Its topology is irregular, with many shallow and narrow channels. Like the remaining states in northeastern Brazil, the border of the shelf ends abruptly (Kempf et al. 1967), at the 60 m isobath between João Pessoa and Recife (Coutinho 1996).

Along the continental shelf of the Paraíba State, bottoms of calcareous algae prevail beyond the isobath of 20 m, with a predominance of the ramified corallinacean alga *Halimeda* Lamoroux, 1812 (Coutinho 1996). Sand and mud bottoms are usually restricted to shallower waters below 20 m, usually occurring as small isolated spots (Kempf et al. 1967).

During the 1980s the Superintendance for the Development of the Northeast (SUDENE) developed the Algae Project along the continental shelfs of the States of Rio Grande do Norte and Paraíba, in order to map and collect banks of calcareous algae in this region. The thoroughest coverage of this project was along the State of Paraíba, and representative samples of ophiuroids were obtained and deposited in the Federal University of that state.

Our aim is to provide a checklist and identification keys for the species of ophiuroids that inhabit the continental shelf of the State of Paraíba, describing species from based on the collected material and summarizing data on their ecology and distribution.

## Materials and methods

The studied material belongs to the Invertebrate Collection Paulo Young, Department of Systematics and Ecology, Federal University of Paraíba (CIPY/DSE–UFPB). Collections were made in 1981 during the Algae Project, on the continental shelf of the State of Paraíba, between coordinates 6°58'S, 34°46'W and 7°34'S, 34°45'W, between the isobaths of 10 and 35 m. The animals were captured with dredges at 93 stations positioned along 23 transects perpendicular to the coast (Fig. 1).

For taxonomic identifications, specimens were dried, observed with a dissecting microscope Olympus SZ40 and identified with the works of A.M. Clark (1953), John and Clark (1954), Fell (1960), Thomas (1973), Tommasi (1970), Manso (1988) and Hendler et al. (1995), Manso et al. (2008), and Benavides-Serrato et al. (2011). The diameter of the disk of each specimen was measured with a digital caliper EDC 6” and photos were obtained with a camera Canon A640 10MP coupled to a stereomicroscope Nikon SMZ800. The mean and standard deviation of the disk diameter were calculated using the software Statistica 7.0. The species names agree with Stöhr and O’Hara (2013) and are arranged systematically following Smith et al. (1995). All material was preserved in ethanol at 70% and deposited in CIPY/DSE. The abundance of species in each collection point is provided on the basis of studied material.

**Abbreviations:** dd–disk diameter. Spec–specimens.

**Acronym:** UFPB.Ech.–Echinodermata Collection of Federal University of Paraíba.

## Results

The fauna of ophiuroids recorded along the shelf of the State of Paraíba contains species known to have a wide geographical and bathymetrical distribution, occurring, in general, along a considerable extension of the coast of Brazil. The number of known species in the State of Paraíba increased from 16 to 23, corresponding to 17% of the Brazilian species and 54% of the northeastern species.

We examined 647 samples (totaling 1.379 specimens), identifying 23 species, 14 genera, 8 families, and 1 order of Ophiuroidea (Tab. 1, Tab. 2, Supplementary Material).

### Checklist of brittle-stars from the continental shelf of the State of Paraíba

Class Ophiuroidea Gray, 1840

Order Ophiurida Müller & Troschel, 1840

Family Ophiomyxidae Ljungman, 1867

*Ophiomyxa flaccida* (Say, 1825)

Family Ophiolepididae Ljungman, 1867

*Ophiolepis impressa* Lütken, 1859

*Ophiolepis paucispina* (Say, 1825)

Family Amphiuridae Ljungman, 1867

*Amphiodia planispina* (von Martens, 1867)

*Amphiodia riisei* (Lütken, 1859)

*Amphipholis januarii* Ljungman, 1866

*Amphipholis squamata* (Delle Chiaje, 1828)

*Amphiura stimpsoni* Lütken, 1859

*Ophiocnida scabriuscula* (Lütken, 1859)

*Ophiophragmus brachyactis* H.L. Clark, 1915

*Ophiostigma isocanthum* (Say, 1825)

Family Ophiotrichidae Ljungman, 1867

*Ophiothrix (Ophiothrix) angulata* (Say, 1825)

Family Ophiactidae Matsumoto, 1915

*Ophiactis quinqueradia* Ljungman, 1872

*Ophiactis savignyi* (Müller & Troschel, 1842)

Family Ophionereididae Ljungman, 1867

*Ophionereis reticulata* (Say, 1825)

*Ophionereis squamulosa* Koehler, 1914

*Ophionereis dolabriformis* John & A.M. Clark, 1954

*Ophionereis olivacea* H.L. Clark, 1900

Family Ophiocomidae Ljungman, 1867

*Ophiocoma echinata* (Lamarck, 1816)

*Ophiocoma wendtii* Müller & Troschel, 1842

*Ophiopsila hartmeyeri* Koehler, 1913

Family Ophiodermatidae Ljungman, 1867

*Ophioderma appressa* (Say, 1825)

*Ophioderma cinerea* Müller & Troschel, 1842

### Key to the families of brittle-stars from the continental shelf of the State of Paraíba

**Table:** 

1	Presence of a clump of dental papillae at the apex of the jaw (Fig. 2h, 10c)	3
–	Without dental papillae	2
2	One pair of infradental oral papillae on apex of jaw (Fig. 4c)	Amphiuridae
–	One apical papilla on apex of jaw (Fig. 9c, 13c)	4
3	A continuous series of oral papillae (Fig. 11c)	Ophiocomidae
–	Without oral papillae (Fig. 2h)	Ophiotrichidae
4	Disk and arms covered with a thick and naked tegument	Ophiomyxidae
–	Disk covered with scales, granules or spines	5
5	Two pairs of bursal slits in each interradius (Fig. 13b, g)	Ophiodermatidae
–	One pair of bursal slits in each interradius (Fig. 3b, 8b, 9b)	6
6	Disk covered only with scales. Oral papillae in continuous series	7
–	Disk covered by scales and spines. Presence of a diastema separating the lateral oral papillae from the apical papillae (Fig. 8b, i)	Ophiactidae
7	Dorsal scales of disk thin and imbricating (Fig. 9a, f, 10a, f). Genital papillae present or absent (Fig. 9b, 10b)	Ophionereididae
–	Dorsal scales of disk thick and imbricating (Fig. 3a). Dorsal arm plate present or absent	Ophiolepididae

### Key to the members of the family Ophiolepididae known from the continental shelf of the State of Paraíba

**Table:** 

1	accessory dorsal arm plates small, restricted to the first segments (Fig. 3a, f). Four to five arm spines	*Ophiolepis impressa*
–	All arm segments with accessory dorsal plates (Fig. 3g, j), except near tip. Two arm spine	*Ophiolepis paucispina*

### Key to the members of the family Amphiuridae known from the continental shelf of the State of Paraíba

**Table:** 

1	Distal oral papilla never opercular	4
–	Distal oral papilla opercular (Fig. 5i, 7j)	2
2	Disk covered by scales. Two perpendicular tentacle scales	3
–	Disk covered by small papillae (Fig. 7f, h). Two almost parallel tentacle scales	*Ophiostigma isocanthum*
3	Three or four arm spines, the second and third with two hyaline denticles at tip (Fig. 5f). Radial shields narrow and long (Fig. 5a)	*Amphipholis januarii*
–	Three arm spines with tip tapering (Fig. 5j). Radial shields slightly longer than broad (Fig. 5g)	*Amphipholis squamata*
4	Oral papillae continuous (Fig. 6g). Two tentacle scales	5
–	Oral papillae separated from infradentals by a gap (Fig. 6c). Single reduced tentacle scale (Fig. 6e)	*Amphiura stimpsoni*
5	Radial shields divergent (like a sheep hoof) (Fig. 6e). Three arm spines slightly flattened	*Ophiocnida scabriuscula*
–	Radial shields contiguous, separated only proximally	6
6	With a fence of broadened papillae on margin of disk (Fig. 7a). Radial shields small and rounded	*Ophiophragmus brachyatis*
–	Without spines on margin of disk	7
7	Primary scales not evident (Fig. 4a). Three arm spines compressed, flattened and blunt	*Amphiodia planispina*
–	Primary scales very evident (Fig. 4f). A well marked row of large scales in interbrachial region. Three short and blunt arm spines, compressed in median and distal region	*Amphiodia riisei*

### Key to the members of the family Ophiactidae known from the continental shelf of the State of Paraíba

**Table:** 

1	With six arms and some spines scattered over the dorsal disk (Fig. 8g). One or two oral papillae (Fig. 8i)	*Ophiactis savignyi*
–	With five arms and some small spines usually restricted to the ventral and lateral sides of the disk (Fig. 8a). Two or three oral papillae	*Ophiactis quinqueradia*

### Key to the members of the family Ophionereididae known from the continental shelf of State of Paraíba

**Table:** 

1	Genital papillae present (Fig. 9b)	2
–	Genital papillae absent (Fig. 10b)	3
2	A distinct line forming a reticulated pattern on the aboral surface of the disk (Fig. 9a). Arm spines markedly compressed, with apex blunt. With dark bands on arm segment separated by three-six light bands (Fig. 9a, d)	*Ophionereis reticulata*
–	Dark blotches on aboral surface of disk. Arm spines markedly compressed. Dark bands occupying two or three arm segments, separated by one light bands	*Ophionereis squamulosa*
3	Sometimes several superposed accessory dorsal arm plate are observed. Three arm spines longer than arm segment (Fig. 10i), match-shaped	*Ophionereis olivacea*
–	Accessory dorsal arm plates small (Fig. 10a, d). Three long, thin spines on arm (Fig. 10d, e)	*Ophionereis dolabriformis*

### Key to the members of the family Ophiocomidae known from the continental shelf of the State of Paraíba

**Table:** 

1	Disk covered by scales and granules (Fig. 11a, e)	2
–	Disk covered only by scales (Fig. 12a). Four to 6 arm spines, ventral spines longest and slightly curved	*Ophiopsila hartmeyeri*
2	Dorsal arm spines robust and broadened (Fig. 11d). Two tentacle scales	*Ophiocoma echinata*
–	Dorsal arm spines long and slender (Fig. 11h). One tentacle scale on first arm segments	*Ophiocoma wendtii*

### Key to the members of the family Ophiodermatidae known from the continental shelf of the State of Paraíba

**Table:** 

1	Radial shields covered by granules (Fig. 13a)	*Ophioderma appressa*
–	Radial shields visible, not covered by granules (Fig. 13f)	*Ophioderma cinerea*

## Systematics

### Family Ophiomyxidae Ljungman, 1867

#### Ophiomyxa flaccida

(Say, 1825)

http://species-id.net/wiki/Ophiomyxa_flaccida

Figure 2a–e , 14a

##### Description.

**Figure 2.**
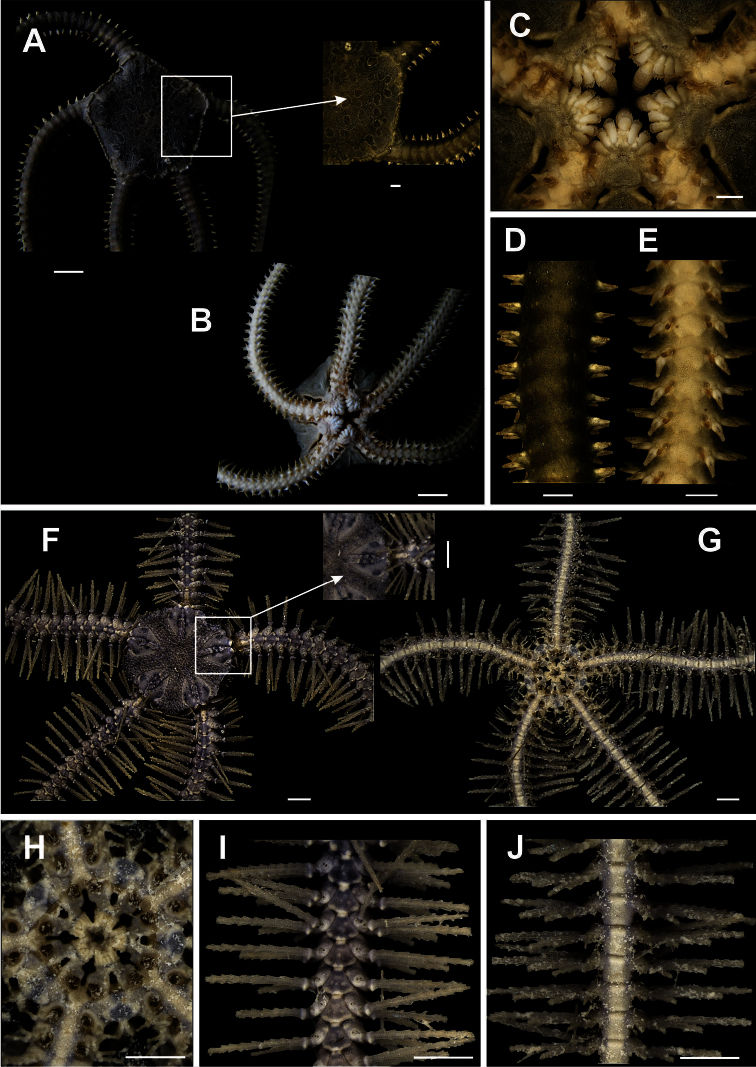
Species of the families Ophiomyxidae (**A–E**) and Ophiotrichidae (**F–J**). *Ophiomyxa flaccida*. **A** dorsal view, in detail the marginal interradius with a row of large scales **B** ventral view **C** jaw **D** dorsal view of the arm **E** ventral view of the arm. *Ophiothrix (Ophiothrix) angulata* **F** dorsal view, in detail the radial shields **G** ventral view **H** jaw **I** dorsal view of the arms **J** ventral view of the arms. Scale bar = 1 mm.

Disk pentagonal (dd = 5.67 to 12.16 mm). Covered by thick and naked tegument (Fig. 2a). Radial shields enlarged along internal margin. Marginal interradius with a row of 8 to 10 large and overlapping scales (Fig. 2a). Bursal slits short and narrow (Fig. 2b). Oral shields triangular to circular, distal margin rounded. Adoral shields narrow and enlarged laterally. Three enlarged oral papillae on each side of jaw angle, distal free end totally dentate, the two proximal papillae being longer and wider than distal papilla (Fig. 2c). Dorsal arm plate long, narrow, fragmented into two (Fig. 2d). Ventral arm plates small, pentagonal, with a small notch on the distal margins. Four to five small, compressed, arm spines, with denticles on tips. Without tentacle scale (Fig. 2e).

##### Distribution.

Bermuda, the islands off southern Florida, the Bahamas, the Antilles, Mexican Caribbean, Belize, Guatemala, Honduras, Panama, islands off Caribbean Colombia, Venezuela, and Brazil (Devaney 1974, Hendler et al. 1995, Chavarro et al. 2004, Durán-Gonzáles et al. 2005, Trujillo-Luna and González-Vallejo 2006, Alvarado et al. 2008, Hernandéz-Herrejón et al. 2008). In Brazil it has been recorded from the States of Amapá, Pará, Maranhão, Ceará, Rio Grande do Norte, Paraíba, Pernambuco, Alagoas (Gondim et al. in press), Abrolhos islands off southern Bahia (Rathbun 1879), Bahia (Alves and Cerqueira 2000), and Rio de Janeiro (Manso 1993). Intertidal to 367.5 m. In this study it occurred between 11 and 33m.

##### Remarks.

According to Hendler et al. (1995), young individuals live associated with the phytal community, while adults are more commonly found on gravel bottoms from seagrass beds. This species feeds ingesting large portions of algae and sponges, detritus being collected by lateral movements of the arms. We observed specimens with stomachs filled with sponge spicules. Although Ophiomyxidae is a taxonomically problematic family (Franklin and O’Hara 2008), with the placement of several genera being uncertain (Martynov 2010). The identification of *Ophiomyxa flaccida* is easy, because the taxonomic characters are relatively constant intraspecifically, except for coloration that can vary between geographic areas (green, yellow, orange, red, reddish with a white spot on the disc or brown). Individuals analyzed in this study had a number of interradial scales smaller than observed by Tommasi (1970) for specimens from southeastern Brazil (about 12 scales). This is apparently a rare species in reef environments of Paraiba, and has been reported only in two reefs of this coast (Gondim et al. 2008, 2011).

### Family Ophiolepididae Ljungman, 1867

#### Ophiolepis impressa

Lütken, 1859

http://species-id.net/wiki/Ophiolepis_impressa

Figure 3a–e , 14b

##### Description.

**Figure 3.**
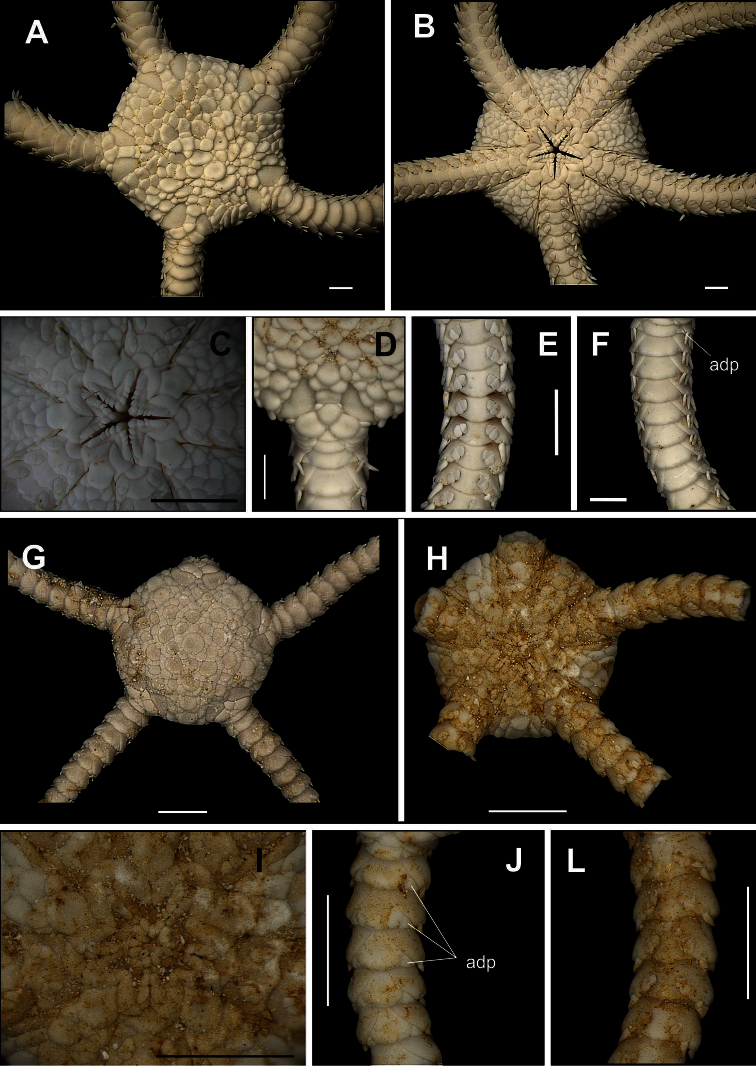
Species of the family Ophiolepididae. *Ophiolepis impressa* **A** dorsal view **B** ventral view **C** jaw **D** detail of the radial shield **E** ventral view of the arms **F** dorsal view of the arms. *Ophiolepis paucispina* **G** dorsal view **H** ventral view **I** jaw **J** dorsal view of the arms (adp: accessory dorsal arm plate) **L** ventral view of the arms. Scale bar = 1 mm.

Disk circular to pentagonal (dd = 4.26 to 9.82 mm). Covered by large, imbricating scales, surrounded by smaller scales of different sizes and irregular shapes (Fig. 3a). Primary plates conspicuous, central primary plate rounded. Interradius with three rows of large scales. Radial shields triangular, separated distally by three large scales disposed in a triangle, and proximally by a large scale (Fig. 3a). Ventral interradius covered by imbricating scales, slightly smaller and narrower than dorsal scales (Fig. 3b). Bursal slits long and narrow. Oral shields pentagonal, elongate, distal margin convex. Adoral shields broad, enlarged laterally and contiguous along internal median line of jaw. Four to five oral papillae on each side of jaw angle, the three proximal of which are pointed and subequal, the penultimate one is longest and broadest (Fig. 3c). Dorsal arm plate wider than long. Accessory dorsal arm plate reduced and restricted to the first arm segments (Fig. 3f). Ventral arm plate on first segments as large as long, on last segments slightly broader than long, tending to become pentagonal in shape, with lateral margins concave and distal margin rounded (Fig. 3e). Three or four arm spines short and conical, blunt, the two dorsal ones smaller. Tentacle pore large. Two large tentacle scales, the outer one slightly broader than the inner one (Fig. 3e).

##### Distribution.

Bermuda, the Bahamas, the islands off southern Florida, Texas, the Antilles, Mexican Caribbean, Belize, Honduras, Costa Rica, Panama, Colombia, Venezuela, and Brazil (Hendler et al. 1995, Laguarda-Figueras et al. 2004, Durán-Gonzáles et al. 2005, Alvarado et al. 2008, Borrero-Pérez et al. 2008, Hernández-Herrejón et al. 2008). In Brazil from Alagoas (Miranda et al. 2012), and Bahia (Tommasi 1970, Magalhães et al. 2005). Intertidal to 24 m in deph. In this study they were found for the first time in the State of Paraíba, between 10 and 33m.

##### Remarks.

According to Hendler et al. (1995), individuals of this species are usually sedentary, but show some nocturnal activity. They live on bottoms with corals and dead shells (Tommasi 1970), predominantly on sand, under corals and rocks, sometimes occurring on algae. They are moderately palatable for some fish, although their strongly calcified arms furnish some protection against predators (Hendler et al. 1995). Only some specimens show a small accessory dorsal arm plate, and when present, it is restricted to the first segments. According to Devaney (1974), Lyman transferred *Ophiolepis impressa* and *Ophiolepis pacifica* Lütken, 1856 to the genus *Ophiozona* Lyman, 1865, based only on the supposed absence of the accessory dorsal arm plate. However, Devaney (1974) rejected the new genus proposed by Lyman and placed it in synonymy with the genus *Ophiolepis*, given that there are no criteria to separate the two taxa. Hendler (1988) questions the use of this character as a synapomorphy of the genus *Ophiolepis*, since it is expressed in different ways. According to this autor, the expression of accessory dorsal arm plates within and between the different species is variable. Moreover, they are barely discernible or absent in juveniles.

#### Ophiolepis paucispina

(Say, 1825)

http://species-id.net/wiki/Ophiolepis_paucispina

Figure 3g–l

##### Description.

Disk circular (dd = 2.74 to 3.55 mm). Covered by large scales, and surrounded by smaller ones of similar size. Central primary plate circular, well defined, surrounded by five small primary plates, and intercalated by two smaller scales (Fig. 3g). Radial shield triangular, separated distally by three large scales disposed in a triangle. Ventral interradius covered by scales, slightly smaller and narrower than dorsal scales (Fig. 3h). Bursal slits long and narrow. Oral shields pentagonal, elongate, distal margin convex (Fig. 3i). Adoral shields broad, enlarged laterally. Four oral papillae on each side of jaw angle, the three proximal of which are pointed and subequal, the last one being longest and broadest (Fig. 3i). Dorsal arm plate fan-shaped (Fig. 3j). Arm segments with accessory dorsal plate, except near the tip (Fig. 3j). Ventral arm plate pentagonal, with lateral margins concave and distal margin rounded. Two tentacle scales oval, outher one larger. Two arm spines small (Fig. 3l).

##### Distribution.

Bermuda, the Bahamas, Florida, Caribbean Sea, Panama, Colombia, Brazil, and off Africa (Hendler et al. 1995, Laguarda-Figueras et al. 2009, Alvarado 2011, Benavides-Serrato et al. 2011, Barboza and Borges 2012). In Brazil, from Alagoas (Lima et al. 2011), and Bahia (Abrolhos) (Magalhães et al. 2005). Intertidal to 37 m in depth (Laguarda-Fiqueras et al. 2009). In this study they were found for the first time in the State of Paraíba, between 30 and 33 m.

##### Remarks.

This species is known from shallow, sandy reef flats, mangrove, lagoonal, and seagrass environments, under coral rubble on sand, in calcareous algae such as *Halimeda*, and among plant debris (Hendler et al. 1995). *Ophiolepis paucispina* is an oviviparous and simultaneous hermaphroditic species (Byrne 1989, Hendler et al. 1995) that broods up to 41 embyos in the genital bursae (Hendler 1979a). In the examined specimens the shape of the adoral shields varied from fan-shape to pentagonal and the number of papillae varied from 3 to 4. It is difficult to separate young individuals of this species from its congener *Ophiolepis impressa*. Yet, the presence of acessory dorsal plates along the entire arm and only two arm spines in *Ophiolepis paucispina* are the most reliable differential characters to distinguish these two species. Personal observations suggest that this is a rare species along the littoral of the State of Paraíba.

### Family Amphiuridae Ljungman, 1867

#### Amphiodia planispina

(von Martens, 1867)

http://species-id.net/wiki/Amphiodia_planispina

Figure 4a–e

##### Description.

**Figure 4.**
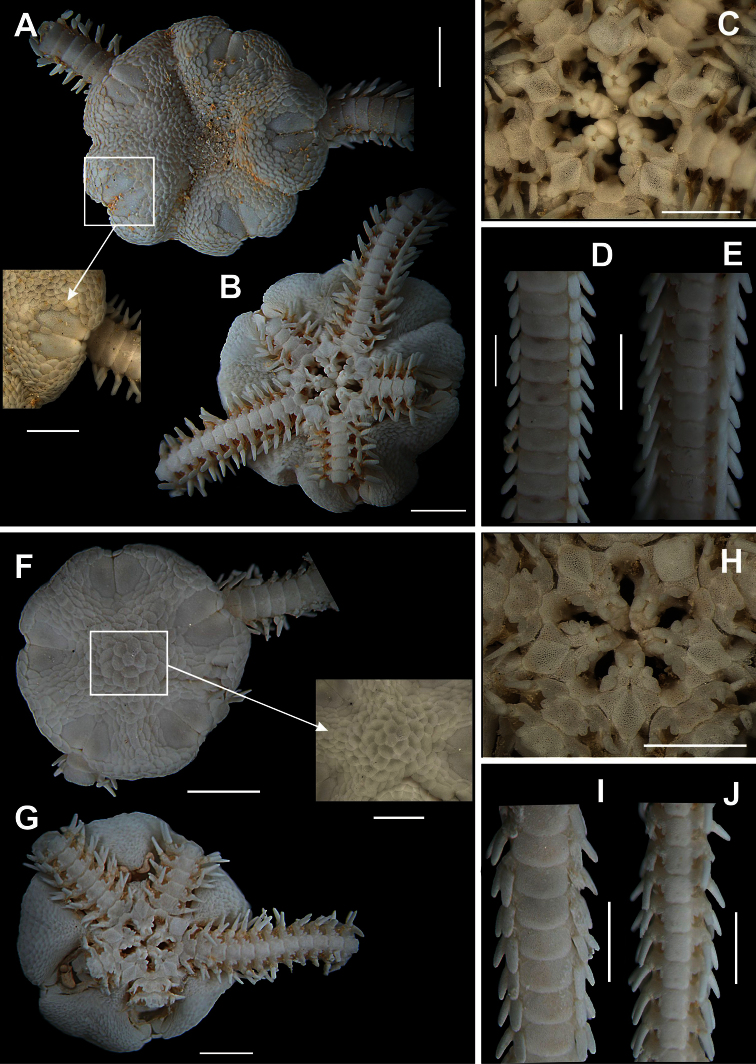
Species of the family Amphiuridae. *Amphiodia planispina* **A** dorsal view, detail of the radial shields **B** ventral view **C** jaw **D** dorsal view of the arms **E** ventral view of the arms. *Amphiodia riisei* **F** dorsal view, detail of the primary plates **G** ventral view **H** jaw **I** dorsal view of the arms **J** ventral view of the arms. Scale bar = 1 mm.

Disk circular (dd= 4.46 to 5.80 mm). Covered by numerous small and imbricating scales (Fig. 4a). Radial shields slightly longer than wide, with external margin convex and internal margin straight, united except at proximal ends, where one or two small triangular and elongate scales separate them (Fig. 4a). Ventral interradius covered by scales slightly smaller than dorsal scales (Fig. 4b). Bursal slits narrow. Oral shields arrow-shaped (Fig. 4c). Madreporite with one or two pores at distal margin. Adoral shields narrow and enlarged laterally. Two oral papillae at each side of jaw angle, distal one longer and broader (Fig. 4c). Infradental papillae small. Dorsal arm plate broader than long, rectangular (Fig. 4d). Ventral arm plate pentagonal, wider than long, with a slight notch in distal margin. Three arm spines enlarged, compressed and blunt, the ventral one being the longest (Fig. 4e). Two small, perpendicular, tentacle scales.

##### Distribution.

Florida, the islands off southern Florida, the Antilles, Panama Brazil, and off Mar del Plata, Argentina (Tommasi 1970, Bernasconi and D’Agostino 1977, Hendler et al. 1995, Alvarado et al. 2008). In Brazil from Maranhão, Ceará, Rio Grande do Norte, Paraíba (Albuquerque 1986), Bahia (Thomas 1962, Magalhães et al. 2005), Espírito Santo (Albuquerque and Guile 1991), Rio de Janeiro (Von Martens 1867, type locality) and São Paulo (Tommasi 1970). Depth 0-300 m. In the present study collected between 11 and 27m.

##### Remarks.

This species is known from bottoms of sand, mud, gravel and algae (Tommasi 1970). It is found burrowed in the sediment together with other ophiuroids such as *Ophiophragmus pulcher* H.L. Clark, 1918 and *Amphioplus albidus* (Ljungman, 1867) in Florida and *Hemipholis elongata* (Say, 1825) in Brazil (Hendler et al. 1995). Thomas (1962) showed that the shape of the arm spines (compressed and blunt) and the noncontiguous adoral shields are important characters to separate *Amphiodia planispina* from the other more closely related species such as *Microphiopholis atra* (Stimpson, 1854). Thomas (1962) remarked that these morphological characters may not be present in all specimens, as was also observed by us. The specimens observed in this study differed from the description provided by Tommasi (1970) only in relation to the number of scales between the radial shields. The specimens (dd = 8.5 mm) analized by Tommasi showed two to seven scales between the radial shields, while the specimens of this study (dd = 5.80 mm) had one or two scales. This fact is probably related to size of the specimens of both studies.

#### Amphiodia riisei

(Lütken, 1859)

http://species-id.net/wiki/Amphiodia_riisei

Figure 4f–j

##### Description.

Disk circular (dd = 4.16 mm). Covered by numerous imbricating scales of irregular shapes (Fig. 4f). Primary plates very conspicuous (Fig. 4f). Central primary plate slightly pentagonal, surrounded by the radial primary plates. Radial shields enlarged distally, contiguous, except at proximal end, where there is a small, elongate, triangular scale (Fig. 4f). Ventral interradius covered by small, imbricating scales (Fig. 4g). Bursal slits long and broadened. Oral shields diamond-shaped (Fig. 4h). Adoral shields enlarged laterally. Two oral papillae on each side of jaw angle, the distal one a little larger than the proximal one (Fig. 4h). Infradental papillae rectangular and robust. Dorsal arm plate broader than long, rectangular, with the distal border rounded (Fig. 4i). Ventral arm plate pentagonal. Three arm spines slightly bigger than one arm segment, which is laterally flattened, with blunt tip (Fig. 4j). Two tentacle scales subequal and perpendicular.

##### Distribution.

Off Florida, the Antilles, possibly Panama and Puerto Rico, and Brazil (Hendler et al. 1995). In Brazil from Amapá, Pará (Albuquerque 1986), Bahia (Magalhães et al. 2005), Rio de Janeiro (Lütken 1859, type locality), São Paulo (Tommasi 1970), and Paraná (Borges and Amaral 2005). Depth 1-311 m. Found for the first time in the State of Paraíba, at 16 m, in the present study.

##### Remarks.

This species is known from sand and mud bottoms (Tommasi 1970). Thomas (1962) suggests that the species may be found in shallow waters, given that several reports collected specimens from 37m. Thomas (1962) suggests that *Ophiophragmus brachyactis* and *Amphiodia riisei* are synonyms. However, we have considered them distinct species belonging to different genera, on the basis of several noted differences, among which we stress the presence of a fence of papillae on the margin of the disk in *Ophiophragmus brachyactis* and the presence of well developed primary plates in *Amphiodia riisei*. In the original description of *Ophiophragmus brachyactis*, based on a specimen of 7 mm in disk diameter (dd), H.L. Clark (1915) did not observe the presence of well developed primary plates, a character emphasized by Tommasi (1970) for individuals of *Amphiodia riisei* with different dd (1.64 to 9 mm). Manso (1988) analysed a specimen of *Ophiophragmus brachyatis* with4 mm in dd and Thomas (1962) examined a specimen with a dd of 8 mm and none of them noticed the presence of well developed primary plates. However, Tommasi (1970) remarked that young individuals of *Amphiodia riisei* may present marginal scales slightly elevated on the border of the disk. In these specimens a single tentacle scale is observed, and the scales on the dorsal surface of the disk, mainly the primary plates, are well developed and elevated, characters that do not agree with the diagnosis of *Ophiophragmus brachyatis*. According to H.L. Clark (1918), one of the most important characters of the genus *Ophiophragmus* is the presence of a fence of papillae along the margin of the disk, although he notes that some species of *Amphiodia* may also have elevated scales on the interbrachial areas, despite their appearance being different from those in *Ophiophragmus*. Thus the proposed synonymy and the combination *Ophiophragmus riisei* proposed by Thomas (1962) and accepted by authors such as Hendler et al. (1995), and Stöhr and O’Hara (2013), are not followed herein. We emphasize that the previously mentioned authors did suggest that this complex of species must be revised.

#### Amphipholis januarii

Ljungman, 1866

http://species-id.net/wiki/Amphipholis_januarii

Figure 5a–f

##### Description.

**Figure 5.**
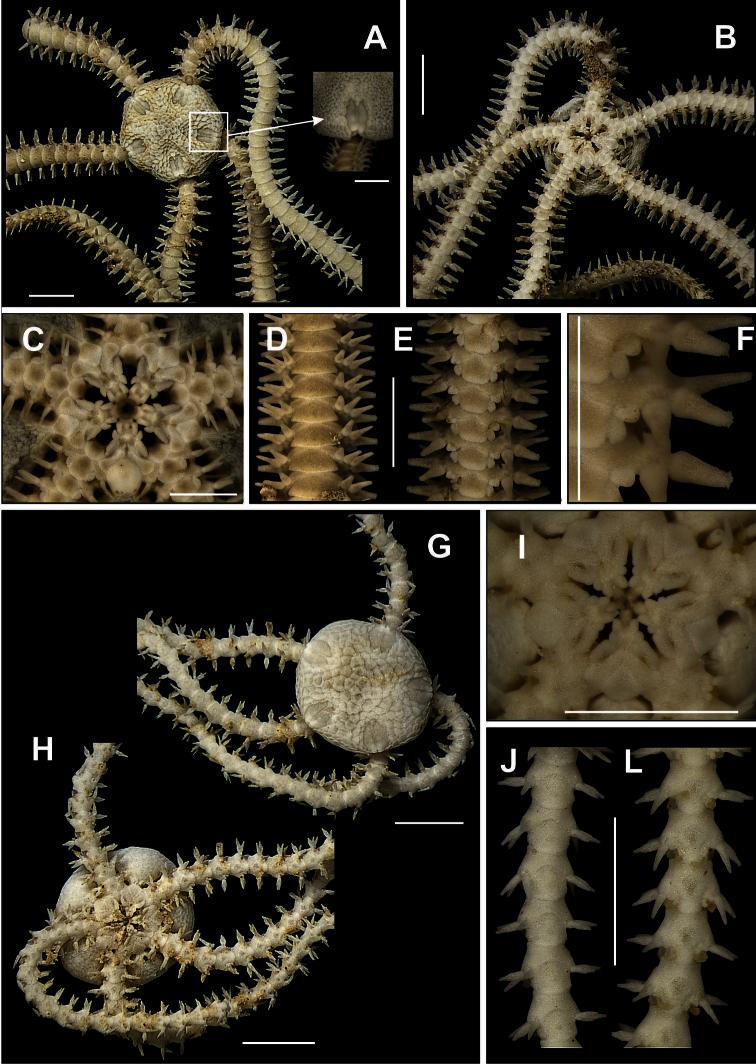
Species of the family Amphiuridae. *Amphipholis januarii* **A** dorsal view, detail of the radial shields **B** ventral view **C** jaw **D** dorsal view of the arms **E** ventral view of the arms **F** detail of the arm spine. *Amphipholis squamata* **G** dorsal view **H** ventral view **I** jaw **J** dorsal view of the arms **L** ventral view of the arms. Scale bar = 1 mm.

Disk circular or pentagonal (dd = 1.90 to 2.70 mm). Covered by small and imbricating scales (Fig. 5a). Radial shields long, narrow, contiguous, separated proximally by a scale (Fig. 5a). Ventral interradius covered by slightly smaller scales, but similar to those on dorsal surface (Fig. 5b). Oral shields diamond-shaped (Fig. 5c). Adoral shields broadened laterally, almost united medially. Two elongated and broadened oral papillae on each side of jaw angle (Fig. 5c). Infradental papillae rectangular and robust. Dorsal arm plate broader than long, with proximal margin rounded and distal margin almost straight (Fig. 5d). Ventral arm plates pentagonal (Fig. 5e). Three to four elongate and blunt arm spines, the second or third with hyaline denticles on tip (Fig. 5f). Two perpendicular tentacle scales, inner scale slightly larger than outer.

##### Distribution.

South Carolina, Florida, the islands off southern Florida, Texas, the Antilles, Belize, Panama, and Brazil (Hendler et al. 1995, Alvarado et al. 2008). In Brazil from Pará (Albuquerque 1986, Borges and Amaral 2005), Ceará (Lima-Verde 1969), Paraíba (Gondim et al. 2008), Alagoas (Lima et al 2011), Bahia (Magalhães et al. 2005), Rio de Janeiro (Ljungman 1867, type locality), and São Paulo (Tommasi 1970). Depth 1 to 85 m. Recorded in this study from 10 to 26 m.

##### Remarks.

Species known from bottoms of mud, sand, shells (Tommasi 1970), between algae, under rocks, tending to be abundant in seagrass beds (Hendler et al. 1995). According to Boffi (1972), juveniles are very abundant in algae.

#### Amphipholis squamata

(Delle Chiaje, 1828)

http://species-id.net/wiki/Amphipholis_squamata

Figure 5g–l

##### Description.

Disk circular (dd = 1.08 to 2.47 mm). Covered by large, irregular, and only slightly imbricating scales (Fig. 5g). Sometimes with the central primary plate evident. Radial scales slightly longer than broad, contiguous, separated proximally by a small scale, with outer margin rounded and inner margin straight. Ventral interradius covered by strongly imbricating scales, which are smaller than dorsal scales (Fig. 5h). Distinct line of demarcation between the scales of the dorsal and ventral surface. Bursal slits long and broad (Fig. 5h). Oral shields fan-shape, distal margin enlarged and convex, slightly longer than wide (Fig. 5i). Adoral shields large, united proximally. Two oral papillae on each side of jaw angle, distal long and opercular (Fig. 5i). A pair of infradental papillae. Dorsal arm plate broader than long, proximal margin rounded and distal margin straight (Fig. 5j). Ventral arm plate pentagonal, twice as long as wide. Three arm spines conical, erect, serrate at tip (Fig. 5l). Two tentacle scales small, narrow and elongated.

##### Distribution.

Traditionally considered cosmopolitan, except for the extreme polar regions (but see remarks). Western Atlantic from Canada, United States, Mexico, the Antilles, Belize, Costa Rica, Panama, Colombia, Brazil, Uruguay, and Santa Cruz Province, Argentina (Bernasconi 1965, Hendler et al. 1995, Hernández-Herrejón et al. 2008, Martínez 2008, Benavides-Serrato et al. 2011). In Brazil, from Pará, Maranhão, Ceará, Paraíba, Alagoas, Bahia (Gondim et al. in press), Rio de Janeiro (H.L. Clark 1915) and, São Paulo (Borges et al. 2002). Intertidal to 1962 m. Found between 21 and 26m in present study.

##### Remarks.

Viviparous polychromatic species, presenting simultaneous hermaphroditism (Nisolle 1990), bioluminescence and fluorescence (Hendler 1996). Commonly found associated with algae and other biological substrates (sponges, cnidarians, bryozoans and molluscs). It may also be found on bottoms of sand, rock, seagrass beds, mangroves, estuaries, and in hypersaline waters. This species has been assigned at least 25 different names (Poulin et al. 1998), now synonymyzed as *Amphipholis squamata*. A.M. Clark (1987) has proposed conservation of this name and the suppression of the older name *Ophiura elegans* (Leach, 1815). *Amphipholis squamata* is regarded as the only species of echinoderm distributed world-wide. This vast distribution area contrasts with a low dispersal potential due to the lack of a pelagic larval stage and an aggregative spatial distribution (Féral et al. 2001). According to Fell (1946), the species extends its distribution by coastal migrations. However, Tortonese (1965) questioned the authenticity of a pandemic species. This species is known to have a strong inter- and intra-population variability among adult individuals for both phenotype and genotype, although the species seems to be anatomically uniform. Dupont et al. (2000) found that polychromatism and bioluminescence might be good indicators of variability of genotypes only at the intra-population level. Féral et al. (2001) confirmed that each color variety possesses its own luminous capabilities and that color varieties are genetically differentiated, although no clear genetic differences were demonstrated between colour varieties. The study of Sponer and Roy (2002) finally confirmed the existence of cryptic species and cryptic dispersal potential in New Zealand. The analysed specimen presented a small variation in the shape of the oral shield (see description) when compared with the specimens described by Thomas (1962), which presented diamond-shaped and wider than long oral shields. Thomas (1962) observed that the vertral surface of the disk was spinulose in two specimens from Puerto Rico (apparently belonging to this species). No specimen from the coast of Paraíba presents this character. According to Hendler (1996), the structure of the arm spines is the most reliable differential character in adult specimens.

#### Amphiura stimpsoni

Lütken, 1859

http://species-id.net/wiki/Amphiura_stimpsoni

Figure 6a–e

##### Description.

**Figure 6.**
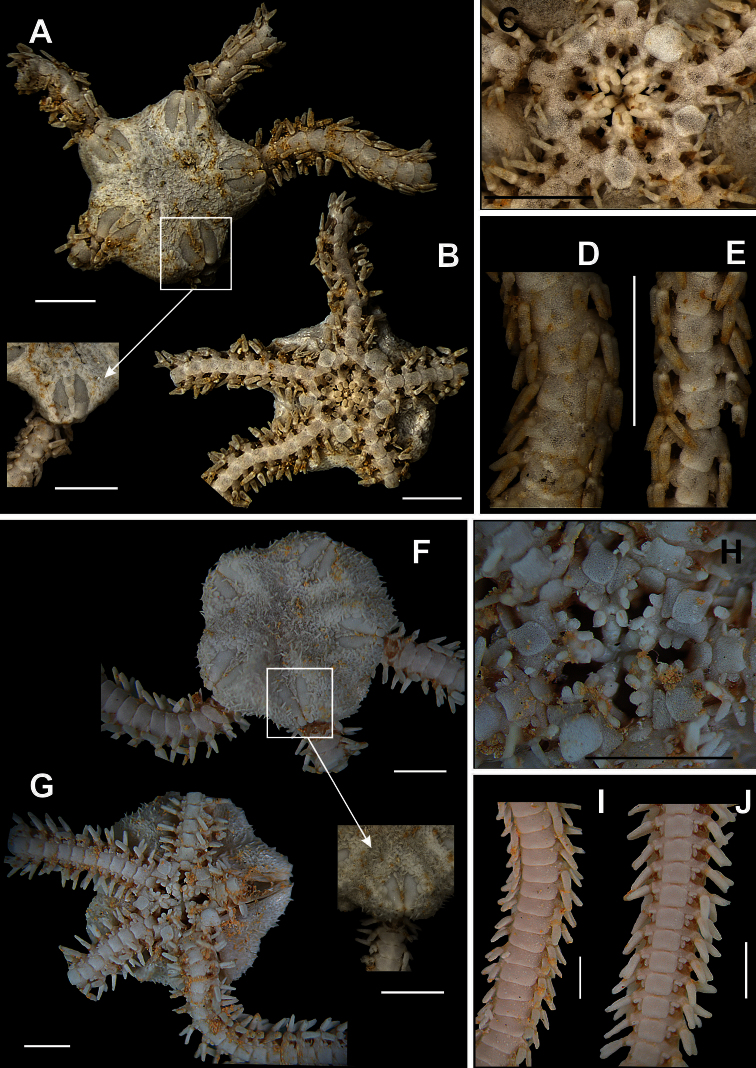
Species of the family Amphiuridae (**A–I**). Amphiura stimpsoni. **A** dorsal view, detail of the radial shields; **B** ventral view **C** jaw **D** dorsal view of the arms **E** ventral view of the arms. *Ophiocnida scabriuscula* **F** dorsal view, detail of the radial shields **G** ventral view **H** jaw **I** dorsal view of the arms **J** ventral view of the arms. Scale bar = 1 mm.

Disk pentagonal (dd = 2.63 to 3.03 mm). Covered by imbricating scales of different sizes (Fig. 6a). Radial shields narrow, three times as long as wide, almost completely separated by two or three broad and elongated scales (Fig. 6a). Ventral interradius covered by scales similar to dorsal, but slightly smaller (Fig. 6b). Bursal slits narrow. Oral shields longer than wide, tending to be diamond-shaped (Fig. 6c). Adoral shields enlarged laterally. Two oral papillae on each side of jaw angle, distal spatuliform and proximal spiniform, the latter positioned more internally on jaw (Fig. 6c). Dorsal arm plate slightly wider than long, proximal angle acute and distal margin rounded, tending to be fan-shaped (Fig. 6d). Ventral arm plate rectangular and narrow (Fig. 6e). Four to five subequal arm spines, crown of denticles on tip. One small tentacle scale (Fig. 6e).

##### Distribution.

The Bahamas, the islands off southern Florida, west coast of Florida, Texas offshore reefs, the Antilles, Belize, islands off Caribbean Colombia, and Brazil (Hendler et al. 1995, Chavarro et al. 2004, Alvarado et al. 2008). In Brazil from Amapá, Maranhão, Ceará, Bahia (Gondim et al. in press), Rio de Janeiro (Rathbun 1879), and São Paulo (Netto et al. 2005). Depth 1 to 126 m. Recorded herein for the first time in the State of Paraíba, between 10 and 18 m.

##### Remarks.

Hermaphroditic and viviparous species. It lives in bottoms of mud, sand, calcareous algae (Tommasi 1970), and gravel of corals and shells (Abreu-Pérez et al. 2005). Two species of genus *Amphiura* are known for the litoral of northeastern Brazil, *Amphiura stimpsoni* and *Amphiura kinbergi* Ljungman, 1872. The latter is recorded only for the states of Alagoas and Bahia (Lima et al. 2011, Manso et al. 2008). *Amphiura stimpsoni* differs formits congener *Amphiura kinbergi* in the number of tentacle scales (one in *Amphiura stimpsoni* and two in *Amphiura kinbergi*) and in the number of arm spines (six to seven in *Amphiura kinbergi*). Personal observations suggest that this species is rare along the littoral of Paraíba, both in shallow coastal waters as in deeper isobates (up to 35 m).

#### Ophiocnida scabriuscula

(Lütken, 1859)

http://species-id.net/wiki/Ophiocnida_scabriuscula

Figure 6e–i

##### Description.

Disk circular with slight indentations in interradius (dd = 4.45 mm). Dorsal and ventral surfaces covered by numerous small spines, also in between the radial shields (Fig. 6e). Scales numerous on disk, imbricating and of different sizes, the largest surrounding the radial shields. Radial shields longer than wide, divergent and with two small accessory plates (Fig. 6e). Bursal slits long. Oral shields arrowhead-shape (Fig. 6g). Adoral shields enlarged laterally. Two oral papillae on each side of jaw angle, small, rounded, and slightly elongated (Fig. 6g). Dorsal arm plate rectangular, narrow, with distal margin rounded (Fig. 6h). Ventral arm plate pentagonal (Fig. 6i). Two small tentacle scales. Three arm spines slightly flattened, the dorsal one slightly longer and wider than the other two.

##### Distribution.

Bermuda, Florida, the islands off southern Florida, the Antilles, Mexican Caribbean, Panama, Colombia, Venezuela, and Brazil (Hendler et al. 1995, Hernández-Herrejón et al. 2008). In Brazil from Maranhão (Albuquerque 1986), Paraíba (Gondim et al. 2008, 2011), Pernambuco (Lima and Fernandes 2009), Alagoas (Lima et al. 2011), Bahia (Rathbun 1879, Magalhães et al. 2005), Abrolhos off southern Bahia, Rio de Janeiro, São Paulo, and Paraná (Tommasi 1970). Intertidal to 68 m. Recorded in this study between 18 and 30 m.

##### Remarks.

This littoral species has a limited bathymetric distribution (H.L. Clark 1933). It is known from hard substrates (sand and gravel) (Tommasi 1970) and from marine seagrasses (Hendler et al. 1995). We observed the formation of a third tentacle scale in some arm segments.

#### Ophiophragmus brachyactis

H.L. Clark, 1915

http://species-id.net/wiki/Ophiophragmus_brachyactis

Figure 7a–e

##### Description.

**Figure 7.**
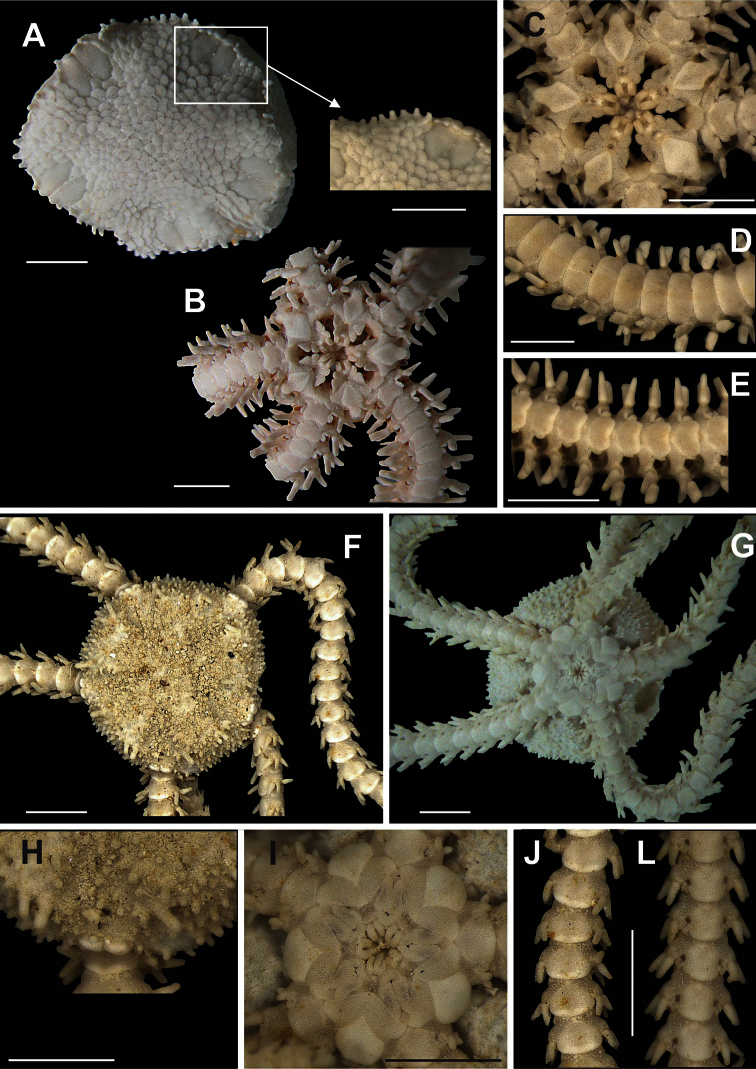
Species of the family Amphiuridae. *Ophiophragmus brachyactis* **A** dorsal view, detail of the fence of papillae **B** ventral view **C** jaw **D** dorsal view of the arms **E** ventral view of the arms. *Ophiostigma isocanthum* **F** dorsal view **G** ventral view **H** detail of the radial shields **I** jaw **J** dorsal view of the arms **L** ventral view of the arms. Scale bar = 1 mm.

Disk circular (dd = 4.45 mm). Covered by imbricating scales of irregular shape (Fig. 7a). Margin of interradial field with 12-13 small, enlarged, blunt spines that decrease in size in the direction of the radial shields (Fig. 7a). Radial shields slightly longer than wide, united, except at proximal end, where a small triangular scale occurs between the pair of radial shields. Ventral interradius covered by scales similar to dorsal scales (Fig. 7b). Oral shields arrow-shaped (Fig. 7c). Adoral shields enlarged laterally, contiguous in proximal region. Two papillae on each side of jaw angle, the distal one longer and wider (Fig. 7c). Infradental papillae robust, and rectangular. Dorsal arm plate wider than long, rectangular, with rounded borders (Fig. 7d). Ventral arm plate pentagonal, with small notch on distal margin (Fig. 7e). Two small, perpendicular, tentacle scales, inner scale slightly longer than outer scale. Three small, compressed, blunt, arm spines, the ventral one largest (Fig. 7e).

##### Distribution.

Florida and Dry Tortugas, the Antilles, Gulf of Mexico, and Brazil (Thomas 1962, Hendler et al. 1995). In Brazil from Paraíba (Gondim et al. 2008), Espírito Santo, and Rio de Janeiro (Manso 1988, 1993). Depth 22 to 87 m. Recorded from 30 m in this study.

##### Remarks.

Species known from bottoms with sand, in which bryozoans predominate (Manso 1988). We consider *Amphiodia riisei* distinct from *Ophiophragmus brachyactis* and use the name *Amphiodia riisei* for the former taxon (see discussion under this species).

#### Ophiostigma isocanthum

(Say, 1825)

http://species-id.net/wiki/Ophiostigma_isocanthum

Figure 7f–l

##### Description.

Disk circular to pentagonal (dd = 1.58 to 3.31 mm). Covered by small blunt tubercles (Fig. 7f). Some large and blunt tubercles distributed in the interradius, usually near the radial shields. Radial shields small (Fig. 7f, h). Ventral interradius covered by short and blunt tubercles similar to the dorsal ones (Fig. 7g). Bursal slits narrow and long. Oral shields fan-shape (Fig. 7i). Adoral shields united proximally and almost touching the adoral shield of the neighbouring jaw along median arm line (Fig. 7i). Two oral papillae on each side of jaw angle, distal operculate, closing oral slit (Fig. 7i). Dorsal arm plate with proximal margin rounded and distal margin straight (Fig. 7j). Ventral arm plate pentagonal, long. Three conical arm spines (Fig. 7l). Two elongate tentacle scale (Fig. 7l).

##### Distribution.

Bermuda, North Carolina to Florida and the island off southern Florida, Texas offshore reefs, the Bahamas, the Antilles, Mexican Caribbean, Panama, Colombia and islands off Caribbean, Colombia, Venezuela, and Brazil (Hendler et al. 1995, Chavarro et al. 2004, Laguarda-Figueras et al. 2004, Durán-Gonzáles et al. 2005, Borrero-Pérez et al. 2008). In Brazil from Pará, Ceará, Paraíba (Albuquerque 1986), Pernambuco (Lima and Fernandes 2009), Alagoas (Albuquerque 1986), Bahia (Magalhães et al. 2005), and Rio de Janeiro (Rathbun 1879). Intertidal to 244 m in depth. Recorded from 14 to 34 m in this study.

##### Remarks.

This species rarely exceeds 7 mm in disk diameter (H.L. Clark 1933). It lives on rocky bottoms, on or in fine sediment, among shell, corals, calcareous algae, under sponges (Tommasi 1970, Hendler 1995, Manso et al. 2008), and among the roots of seagrasses (Thomas 1962). This is a cryptic species that hides by burrowing into the sediment. Individuals with 1mm in disk diameter may already have mature gonads (Hendler et al. 1995). In specimens having the disc in the process of regenerating, only small granules were observed distributed over the whole disc, the large granules being absent from the inter-radial regions.

### Family Ophiotrichidae Ljungman, 1867

#### Ophiothrix (Ophiothrix) angulata

(Say, 1825)

http://species-id.net/wiki/Ophiothrix_angulata

Figure 2f–j , 14c

##### Description.

Disk circular (dd = 0.63 to 4.79 mm). Covered by small, hyaline bifid or trifid spines, also on the radial shields (Fig. 2f). Radial shields longer than wide, separated by a row of scales (Fig. 2f). Ventral interradius covered by spines similar to dorsal (Fig. 2g). Bursal slits short and wide. Oral shields enlarged laterally, triangular, with distal margin (Fig. 2h). Adoral shields united proximally. No oral papillae, but jaws bear terminal clump of dental papillae (Fig. 2h). Dorsal arm plate fan-shaped (Fig. 2i). Ventral arm plate slightly longer than wide, hexagonal, with distal margin long and slightly concave (Fig. 2j). Nine long arm spines, vitreous and denticulate, the one but last smallest and the last modified into a hook. Single tentacle scale small.

##### Distribution.

Bermuda, North Carolina to Texas coast and offshore reefs, Dry Tortugas, the Bahamas, the Antilles, Mexican Caribbean, Honduras, Belize, Panama, islands off Caribbean, Colombia, Venezuela, Brazil, Uruguay, and off La Plata river, Argentina (Tommasi 1970, Devaney 1974, Hendler et al. 1995, Chavarro et al. 2004, Durán-Gonzáles et al. 2005, Laguarda-Figueras et al. 2005, Alvarado et al. 2008, Borrero-Pérez et al. 2008, Hernandéz-Herrejón et al. 2008, Martínez 2008). In Brazil from Amapá (Albuquerque 1986), Piauí (Gondim and Giacometti 2010), Paraíba (Rathbun 1879), Pernambuco (Tommasi 1970), Alagoas (Miranda et al. 2012), Bahia (Alves and Cerqueira 2000), Abrolhos off southern Bahia, Trindade oceanic island off Espírito Santo (Tommasi 1970), Rio de Janeiro (Rathbun 1879), São Paulo, Paraná, Santa Catarina, and Rio Grande do Sul (Tommasi 1970). Intertidal to 540 m depth. Sampled between 10 and 34 m depth in this study.

##### Remarks.

Associated with seaweeds, such as the brown alga *Sargassum* spp. (Jacobucci et al. 2006), living between stones, and in sponges (Tommasi 1967), in oyster banks, mangroves, seagrass beds and on sessile animals such as *Millepora* sp. and gorgonians (Hendler et al. 1995). Also reported in Brazil from colonies of the octocoral *Carijoa riisei* (Neves et al. 2007), from the tubes of the polychaete *Phyllochaetopterus socialis* Claparède, 1870 (Nalesso et al. 1995), in the sponge *Zygomycale parishii* (Bowerbank, 1875) (Duarte and Nalesso 1996), and from colonies of the bryozoan *Schizoporella errata* (Walters, 1878) (Morgado and Tanaka 2001). Individuals with a disk diameter smaller than 4.0 mm do not have long spines on the median region of the dorsal disk surface (Monteiro 1987). This is a common and highly variable species, with planktotrophic larvae (Hendler 2005). It displays great variation in color, Tommasi (1970) listed 21 different color forms of this species (Hendler et al. 1995). On the coast of Paraíba, the most commonly observed color is violet or specimens which are violet only on the disk and have aniline-blue arms.

### Family Ophiactidae Matsumoto, 1915

#### Ophiactis quinqueradia

Ljungman, 1872

http://species-id.net/wiki/Ophiactis_quinqueradia

Figure 8a–f

##### Description.

**Figure 8.**
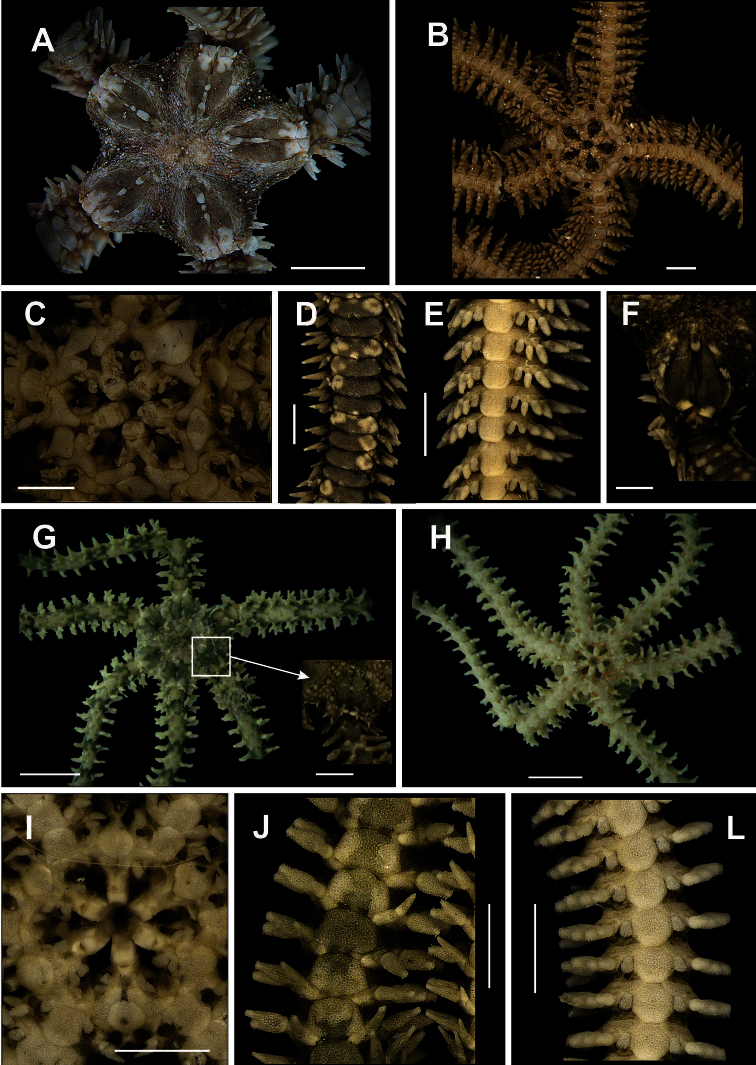
Species of the family Ophiactidae. *Ophiactis quinqueradia* **A** dorsal view **B** ventral view **C** jaw **D** dorsal view of the arms **E** ventral view of the arms **F** detail of the radial shields. *Ophiactis savignyi* **G** dorsal view, detail of the radial shields **H** ventral view **I** jaw **J** dorsal view of the arms **L** ventral view of the arms. Scale bar = 1 mm.

Five arms. Disk circular to pentagonal (dd = 2.48 to 7.62 mm). Covered by imbricating scales of irregular sizes. Scales at center of disk smaller, the largest on interradial field and mainly on lateral margins of radial shields (Fig. 8a). Sparsely distributed small spines on aboral region of disk. Radial shields long and separated over almost full length by three long scales (Fig. 8f). Ventral interradius covered by small spines on small and imbricating scales (Fig. 8b). Bursal slits long and wide. Oral shields long, diamond-shaped, proximally enlarged and distally narrow (Fig. 8c). Adoral shields large, laterally wide. Two or three small, spatulate, oral papillae, distal papilla broader and proximal papilla slightly curved towards the interior of the mouth (Fig. 8c). Dorsal arm plate wider than long, rectangular, with proximal margin rounded (Fig. 8d). Ventral arm plate hexagonal (Fig. 8e). Five or seven serrated arm spines, with a crown of denticles at tip. First dorsal spine small, second largest, and remaining decrease in size in the ventral direction. Single tentacle scale spatulate (Fig. 8e).

##### Distribution.

The Bahamas, the islands off southern Florida, off Mississippi, Texas offshore reefs, the Antilles, Mexican Caribbean, Cuba, Belize, Panama, and Brazil (Hendler et al. 1995, Durán-Gonzáles et al. 2005, Hernández-Herrejón et al. 2008). In Brazil from Maranhão, Ceará, Rio Grande do Norte, Pernambuco, Alagoas (Albuquerque 1986, Gondim et al. *in press*), and Espírito Santo (Tommasi 1970). This is the first record for the State of Paraíba. Intertidal to 640 m. Recorded between 11 and 34 m in the present study.

##### Remarks.

This species lives on bottoms of mud, sand, gravel or corals, being very common in sponges (Tommasi 1970). According to Hendler et al. (1995), it is an endocommensal of sponges. In Cuba, concentrations of 200 to 300 individuals of *Ophiactis quinqueradia* were recorded as endocommensal of sponges *Agelas* sp. (Abreu-Pérez et al. 2005), *Verongia lacunosa* (Lamarck, 1816) and *Neofibularia notitangere* (Duchassaing and Michelotti, 1864) (Hopkins et al. 1977, Kissling and Taylor 1977, Hendler 1984). In the examined specimens a dorsal arm plate was sometimes subdivided into two or three small plates of irregular shapes.

#### Ophiactis savignyi

(Müller & Troschel, 1842)

http://species-id.net/wiki/Ophiactis_savignyi

Figure 8g–l

##### Description.

Six arms. Disk circular (dd = 1.3 mm). Covered by numerous imbricating scales. Some small spines on scales at margin of disk (Fig. 8g). Radial shields large, triangular, contiguous (Fig. 8g). Ventral interradus with some spines on the scales (Fig. 8h). Bursal slit enlarged. Oral shields oval (Fig. 8i). Adoral shields widened laterally. One oral papilla on each side of jaw angle (Fig. 8i). Apical papilla well developed. Dorsal arm plate wider than long, sometimes subdivided into two plates (Fig. 8j). Ventral arm plate octogonal. Six arm spines, with denticles along margin and at tip (Fig. 8l). Single tentacle scale semi-elliptical (Fig. 8l).

##### Distribution.

Cosmopolitan, in warm waters throughout the western Indo-Pacific, eastern Pacific, including Malpelo Island off western coast of Colombia, and both sides of the Atlantic, including Ascension island in the South Atlantic. Western Atlantic from South Carolina, Bermuda, Mexican Caribbean, Honduras, and Brazil (Devaney 1974, Pawson 1978, Hendler et al. 1995, Durán-Gonzáles et al. 2005, Cohen-Rengifo et al. 2009). In Brazil from Amapá, Pará, Maranhão (Albuquerque 1986), Ceará (Lima-Verde 1969), Paraíba (Gondim et al. 2008), Pernambuco (Tommasi 1970), Alagoas (Miranda et al. 2012), Bahia (Alves and Cerqueira 2000), Abrolhos off southern Bahia (Tommasi 1970), Rio de Janeiro (Brito 1960), and São Paulo (Tommasi 1970). Intertidal to 518 m. Found at 10 m in this study.

##### Remarks.

Species found in all reef zones, seagrass beds, mangroves, and in contaminated communities (Hendler et al. 1995). According to Madsen (1970), *Ophiactis savignyi* is very polymorphic, resulting in a vast synonymy. Juveniles are frequently found in large densities inside sponges, possibly as commensals (Hyman 1955). Cuénot (1948) considers this behavior a case of pseudocommensalism, due to its marked positive stereotropism. Young forms (up to 4 mm in disk diameter) reproduce by fissiparity, although Devaney (1974) found no indications of this reproductive mode, while large specimens may reproduce both sexually and asexually (Tommasi 1970). Gondim et al. (2008) observed that they may live permanently in phytal communities, in which several life stages were found.

### Family Ophionereididae Ljungman, 1867

#### Ophionereis reticulata

(Say, 1825)

http://species-id.net/wiki/Ophionereis_reticulata

Figure 9a–e

##### Description.

**Figure 9.**
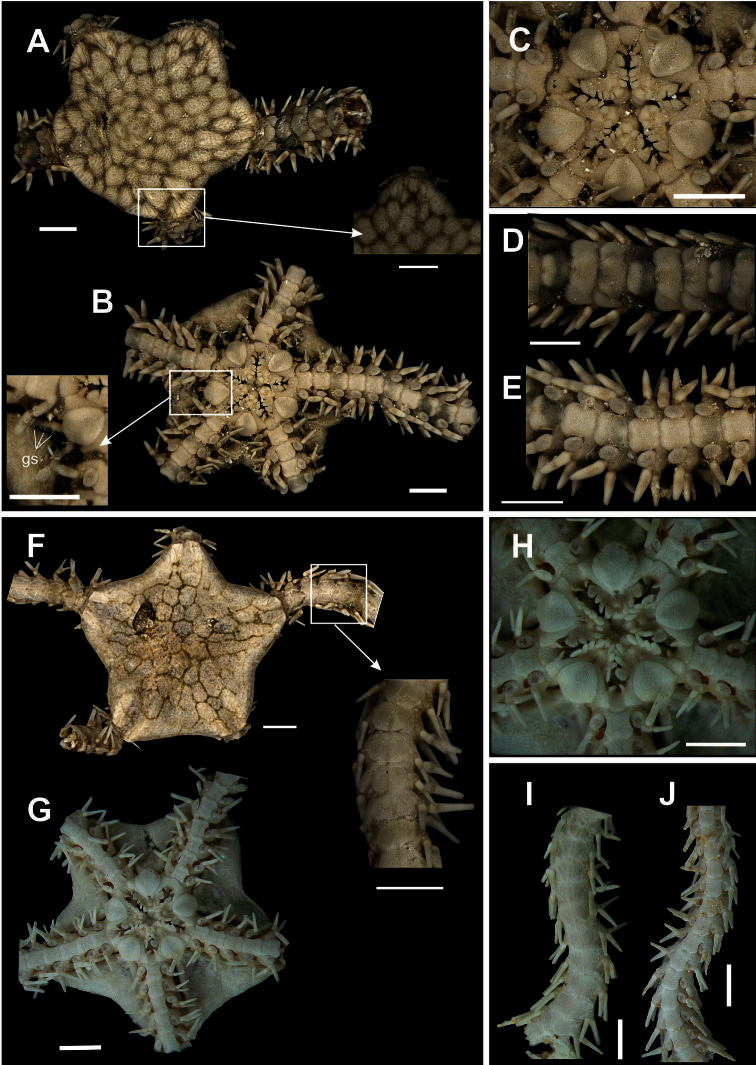
Species of the family Ophionereididae. *Ophionereis reticulata* **A** dorsal view, detail of the radial shields **B** ventral view, detail of the genital scale (gs) **C** jaw **D** dorsal view of the arms **E** ventral view of the arms. *Ophionereis squamulosa* **F** dorsal view, detail of the accessory dorsal arm plate (adp) **G** ventral view **H** jaw **I** dorsal view of the arms **J** ventral view of the arms. Scale bar = 1 mm.

Diskcircular to pentagonal (dd = 1.94 to 6.59 mm). Covered by numerous small and imbricating scales (Fig. 9a). Radial shields small, triangular, elongated and largely separated (Fig. 9a). Aboral surface of disk finely reticulated by fine brownish lines (Fig. 9a). Bursal slits large and with genital papillae (Fig. 9b). Oral shields diamond-shape, longer than wide (Fig. 9c). Adoral shields distally flaring. Three to five oral papillae on each side of jaw angle (Fig. 9c). Distal oral papilla slightly larger, other papillae diminishing progressively in size towards the mouth. Two to three apical papillae. Dorsal arm plates as wide as long, with rounded borders (Fig. 9d). Accessory dorsal arm plates well developed, not touching the neighbouring dorsal plates. Three slightly flattened spines on lateral arm plates (Fig. 9e). Single large, rounded, tentacle scale. Dark brown band (same colour as disk reticulation) along one arm segment, alternated by 3-6 light bands (Fig. 9d).

##### Distribution.

Bermuda, North Carolina, South Carolina offshore reefs, Florida and the islands off southern Florida, the Bahamas, Texas offshore reefs, the Antilles, Mexican Caribbean, Belize, Honduras, Costa Rica, Panama, coast and islands off Caribbean Colombia, Venezuela, and Brazil (Hendler et al. 1995, Chavarro et al. 2004, Durán-Gonzáles et al. 2005, Alvarado et al. 2008, Hernández-Herrejón et al. 2008). In Brazil from Maranhão (Albuquerque 1986), Paraíba (Gondim et al. 2008), Pernambuco (Fernandes et al. 2002), Alagoas (Miranda et al. 2012), Bahia (Brito 1962), Abrolhos, off southern Bahia (Tommasi 1970), Rio de Janeiro (Brito 1960) and São Paulo (Brito 1962). Intertidal to 560 m. Found between 10 and 33 m in this study.

##### Remarks.

Occurs in moderate densities in seagrass beds, and on sand with pebbles (Hendler et al. 1995). It is known for its cannibalistic and predatory habit (Majer et al. 2009). *Ophionereis reticulata* is common in shallow waters, living in reef zones under rocks, in coral fragments, and among algae. It has nocturnal habits. Autotomy is frequent (Ventura et al. 2007). It displays negative phototaxy, prefering dark crevices (Borges and Amaral 2005). The species is omnivorous, but feeding mainly on food of vegetable origin (Yokoyama and Amaral 2008). The ambulacral feet are used to dig, to maintain a flow of particles for feeding, including algae and diatom filaments from the surface sediment, and for locomotion (Hendler et al. 1995). The moderately large eggs suggest that this species has a lecithothrophic development (Hendler and Littman 1986). *Ophionereis reticulata* has been recorded in commensal association with the polychaete *Malmgreniella variegata* (Treadwell, 1917) (Pettibone 1993, Santa-Isabel et al. 1996, Martin and Britayev 1998), and *Hesione picta* (De Assis et al. 2012). This worm-brittle star symbiosis has also been reported for *Ophionereis annulata* (Le Conte, 1851) in the Gulf of Panama, suggesting that the association predates the Pliocene separation of the Atlantic and Pacific oceans (Hendler et al. 1995). According to Clark (1953) this species is closely related to *Ophionereis annulata* (Le Conte, 1851), which differs as to the length of the arm spine, aspect of the dorsal arm accessory plate, and reticulate pattern of the disc. The reticulate pattern is a taxonomic character widely used in distinguishing among the species of the genus *Ophionereis*. Unfortunately the material studied has lost much of this information. But the characters of the arms were important in distinguishing species.

#### Ophionereis squamulosa

Koehler, 1914

http://species-id.net/wiki/Ophionereis_squamulosa

Figure 9f–j

##### Description.

Disk circular (dd = 2.46 to 5.33 mm). Covered by numerous small and imbricating scales (Fig. 9f). Radial shields small, narrow and widely separated. Dark blotches on aboral surface of disk. Disk scales extending onto first brachial segment. Ventral interradius covered by scales similar to dorsal ones (Fig. 9g). Oral shields oval (Fig. 9h). Adoral shields enlarged laterally. Four oral papillae on each side of jaw angle (Fig. 9h). One pair of apical papillae. Dorsal arm plates longer than wide, proximal margin enlarged and distal margin narrow. Accessory dorsal arm plates well developed (Fig. 9f, i). Ventral arm plates slightly longer than wide (Fig. 9j). Three arm spines slightly flattened, with blunt tip (Fig. 9i, j). Single large tentacle scale.

##### Distribution.

The Bahamas, the islands off southern Florida, the Antilles, Mexican Caribbean, Belize, Panama, and Brazil (Hendler et al. 1995, Abreu-Pérez et al. 2005, Trujillo-Luna and González-Vallejo 2006, Alvarado et al. 2008). In Brazil from Amapá (Albuquerque 1986), Paraíba (H.L. Clark 1915), Pernambuco (Albuquerque 1986), Alagoas (Miranda et al. 2012), Bahia (Manso et al. 2008), Abrolhos off southern Bahia (Tommasi 1970), and Rio de Janeiro (Manso 1993). From 1 to 40 m. Recorded between 12 and 30 m in this study.

##### Remarks.

Known from bottoms of sand, gravel, dead shells (Tommasi 1970), and seagrass beds. This species has yolky, non-feeding vitellaria larvae (Hendler et al. 1995). Although similar to *Ophionereis reticulata*, *Ophionereis squamulosa* differs in color-pattern, maximum size, and often size and shape of the accessory dorsal arm plates (Thomas 1973). Yet according to Thomas (1973), *Ophionereis squamulosa* has a difuse, poorly delineated reticulate pattern on the disk and light arm bands separed by a single arm segments. Unfortunately our material is do not retained such features, being not possible to accurately observe their reticulate pattern of the disc.

#### Ophionereis dolabriformis

John & A.M. Clark, 1954

http://species-id.net/wiki/Ophionereis_dolabriformis

Figure 10a–e

##### Description.

**Figure 10.**
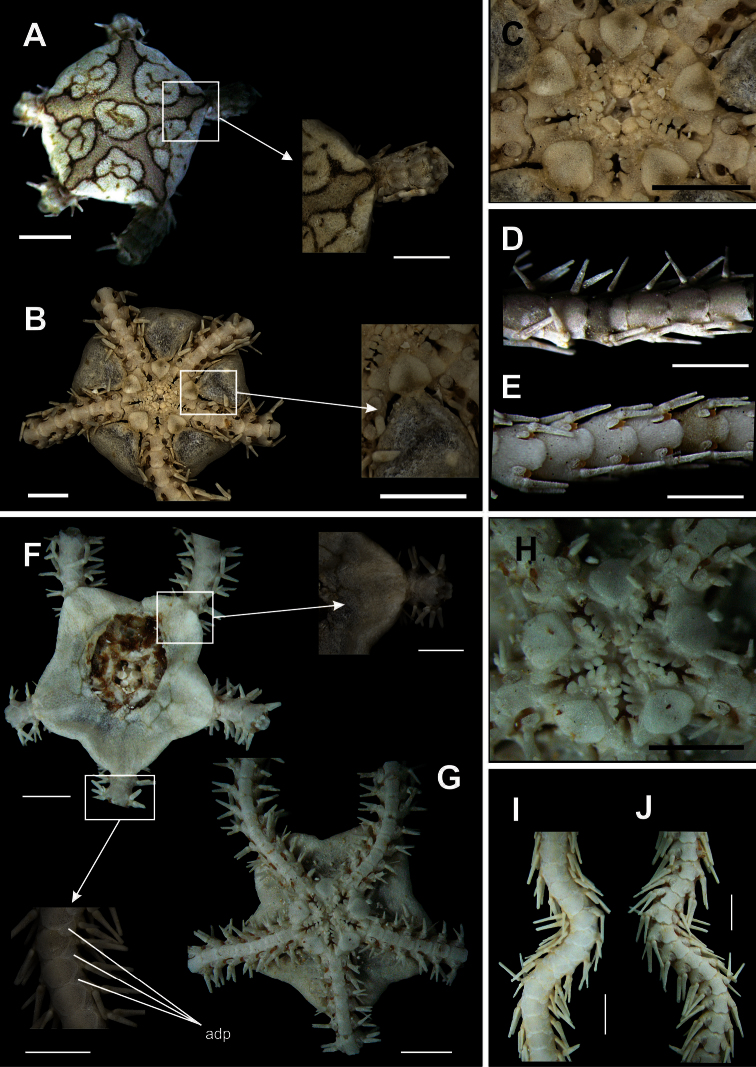
Species of the family Ophionereididae. *Ophionereis dolabriformis* **A** dorsal view, detail of the radial shields **B** ventral view, detail of the genital scale (gs) **C** jaw **D** dorsal view of the arms **E** vental view of the arms. *Ophionereis olivacea* **F** dorsal view, detail of the radial shields **F** ventral view, detail of the accessory dorsal arm plate (adp) **H** jaw **I** dorsal view of the arms **J** ventral view of the arms. Scale bar = 1 mm.

Disk circular to pentaradial (dd = 3.20 to 5.09 mm). Covered dorsally by imbricating scales of different sizes (Fig. 10a). Radial shields small, narrow, triangular (Fig. 10a). Pentaradial olive-green colour pattern on dorsal surface of disk (Fig. 10a). Bursal slits long and without genital papillae (Fig. 10b). Oral shields arrowhead-shaped, partially covering the adoral shields (Fig. 10c). Adoral shields united proximally and enlarged distally. Four oral papillae on each side of jaw angle (Fig. 10c). Dorsal arm plate longer than wide, distal region strongly convex (Fig. 10d). Ventral arm plate rectangular, lateral margins concave, distal margin enlarged and slightly convex (Fig. 10e). Single large, oval, tentacle scale. Three elongate arm spines, needle-shaped (Fig. 10d, e), fully denticulate. Olive-green stripe on 1 to 2 ½ dorsal arm segments.

##### Distribution.

Caribbean coast of Mexico and Colombia, Venezuela (A.M. Clark 1953, Thomas 1973, Pomory 2007, Benavides-Serrato et al. 2011), and Brazil, from Paraíba and Bahia (Gondim et al. 2010). From 14 to 97 m. Found in this study between 14 and 35 m.

##### Remarks.

*Ophionereis dolabriformis* seems to be a rare species, with a high tolerance of river influence (Gondim et al. 2010). Gondim et al. (2010) noted that this species may present variations in the design of the disc patterns, but the pentaradial pattern was maintained, and could be completely uniform, or empty, or with the pattern rays being connected by ﬁne lines.

#### Ophionereis olivacea

H.L. Clark, 1900

http://species-id.net/wiki/Ophionereis_olivacea

Figure 10f–j

##### Description.

Disk usually pentagonal (dd = 3.15 to 3.75 mm). Covered by small and imbricating scales (Fig. 10f). Radial shields small, triangular, narrow, elongated and broadly separated (Fig. 10f). Largest scales surrounding and in between radial shields. Ventral interradius covered by imbricating scales similar to dorsal ones (Fig. 10g). Oral shields tending to heart-shape (Fig. 10h). Adoral shields enlarged laterally. Four oral papillae on each side of jaw angle (Fig. 10h). Dorsal arm plate slightly longer than wide (Fig. 10i). Accessory dorsal arm plate small, sometimes with several overlapping plates (Fig. 10f, i). Ventral arm plate slightly longer than wide (Fig. 10j). Three arm spines slightly larger than arm segment. Single large tentacle scale.

##### Distribution.

The Florida Keys, the Antilles, the Mexican Caribbean, Belize, Panama, the Colombian Caribbean (Hendler et al. 1995, Abreu-Pérez et al. 2005, Alvarado et al. 2008), and Brazil, from Pará (Albuquerque 1986), and Rio de Janeiro (Manso 1993). Intertidal to 77m. Sampled for the first time in the State of Paraíba, between 18 and 30 m, in this study.

##### Remarks.

Known from bottoms of quartz sand, corals, coral fragments, mangroves, and phytal communities (Hendler et al. 1995). This is a protandric hermaphrodite (Byrne 1991) that broods its young (Hendler and Littman 1986). The ciliated embryo lacks both ophiopluteus and vitellaria features and develops directly (Hendler et al. 1995). According to Hendler et al. (1995) this species has a gray disk, with gray-green blotches and an irregular dense or netlike pattern of the same color.

### Family Ophiocomidae Ljungman, 1867

#### Ophiocoma echinata

(Lamarck, 1816)

http://species-id.net/wiki/Ophiocoma_echinata

Figure 11a–e , 14d

##### Description.

**Figure 11.**
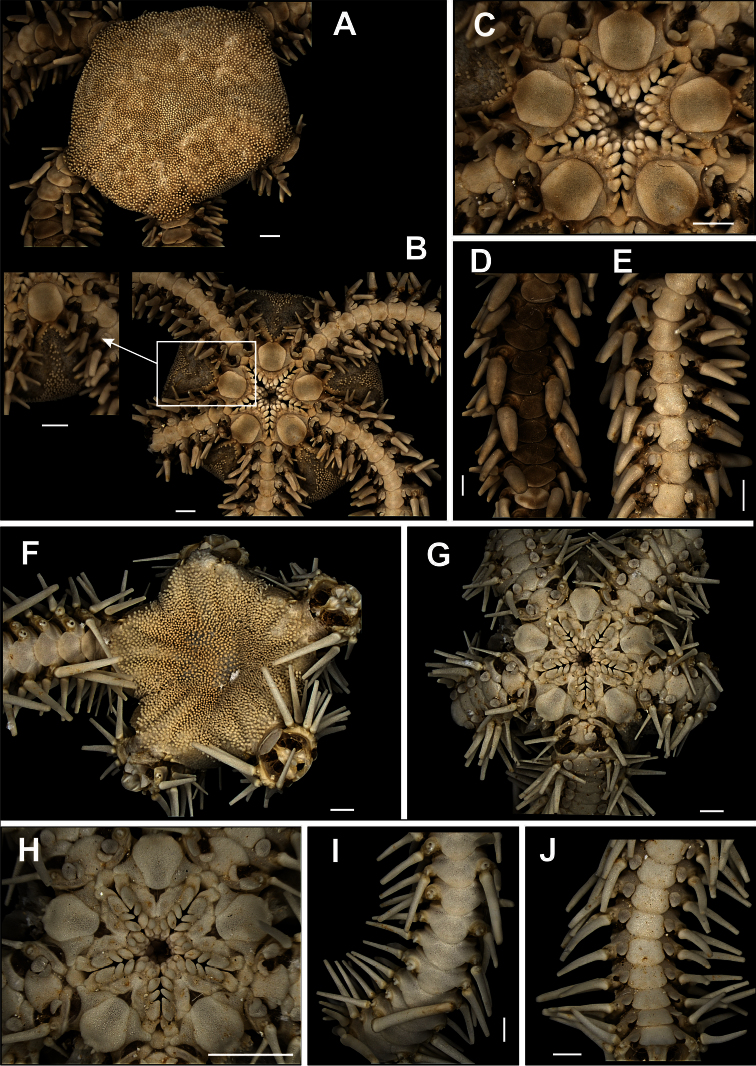
Species of the family Ophiocomidae. *Ophiocoma echinata* **A** dorsal view **B** ventral view, detail of the genital scale (gs) **C** jaw **D** dorsal view of the arms **E** ventral view of the arms. *Ophiocoma wendtii* **F** dorsal view **G** ventral view **H** jaw **I** dorsal view of the arms **J** ventral view of the arms. Scale bar = 1 mm.

Disk circular to pentagonal (dd = 3.06 to 16.68 mm). Uniformly covered by small granules (Fig. 11a), which are smaller in central region than in marginal region. These granules occupy a v-shaped area on the ventral interradius (Fig. 11b). In the areas without granules there are large and imbricating scales. Bursal slits enlarged, with well developed genital scales in margins (Fig. 11b). Oral shields large and rectangular, proximal margin slightly rounded (Fig. 11c). Adoral shields small, almost totally covered by oral shield. Four oral papillae on each side of jaw angle (Fig. 11c). Two proximal papillae slightly cylindrical and subequal, two distal papillae longer and broader. Cluster of well developed dental papillae on apex of jaw (Fig. 11c). Dorsal arm plate longer than wide, fan-shaped (Fig. 11d). Ventral arm plate longer than wide, octogonal, with distal margin slightly convex (Fig. 11e). Two tentacle scales, internal one slightly larger than external one. Three or four arm spines alternating on arm segments. Dorsal spine longer and broader (bottle-shaped) (Fig. 11d), median ones of equal size and ventral one smaller and slightly flattened.

##### Distribution.

Bermuda, Florida and the islands off southern Florida, the Bahamas, the Antilles, the Mexican Caribbean, Belize, Nicaragua, Guatemala, Honduras, Costa Rica, Panama, Colombia, Venezuela, and Brazil (Lyman 1865, H.L. Clark 1933, Hendler et al. 1995, Durán-Gonzáles et al. 2005, Alvarado et al. 2008). In Brazil from Ceará (Albuquerque 1986), Paraíba (Rathbun 1879), Pernambuco (Tommasi 1970), Alagoas (Miranda et al. 2012), Bahia (Tommasi 1970), and Rio de Janeiro (Manso 1993). Intertidal to 24m. Recorded herein between 10 and 34m.

##### Remarks.

This species has diurnal habits. It lives in reef zones, seagrass beds, mangroves, being particularly abundant under rocks (Hendler et al. 1995). It is frequently recorded together with *Ophiocoma pumila* Lütken, 1856, *Ophiocoma wendtii* and *Ophioderma appressa*, although it has an agressive defensive reaction and competes for space with *Ophiocoma wendtii* (Side and Woodley 1985).

#### Ophiocoma wendtii

Müller & Troschel, 1842

http://species-id.net/wiki/Ophiocoma_wendtii

Figure 11e–i

##### Description.

Disk pentagonal with small notches on interradius (dd = 2.71 to 15.07 mm) (Fig. 11e). Covered by small, imbricating scales, totally covered by small granules that extend over the first three dorsal arm segments (Fig. 11e). Ventral interradius covered by granules, that form a V-shaped area (Fig. 11f). Bursal slits long and enlarged. Oral shields triangular (Fig. 11g). Adoral shields distally flaring, not touching medially. Four oral papillae on each side of jaw angle (Fig. 11g), the one but last largest and partially covering last papilla. A cluster of papillae on jaw apex (Fig. 11g). Dorsal arm plate wider than long, fan-shaped (Fig. 11h). Ventral arm plate pentagonal, with distal margin rounded (Fig. 11i). Single large tentacle scale, but the six first arm segments may present two scales. Three or four long, pointed, arm spines, with blunt tip, the dorsal one biggest, four or five arm segments long (Fig. 11h). They decrease in size in the direction of the ventral spine, which is slightly curved. Arm segments with three or four alternating spines.

##### Distribution.

Bermuda, the Bahamas, the islands off southern Florida, Texas offshore reefs, the Antilles, Mexican Caribbean, Belize, Honduras, Costa Rica, Panama, islands off Caribbean Colombia, Venezuela, and Brazil (Lyman 1865, H.L. Clark 1933, Hendler et al. 1995, Chavarro et al. 2004, Durán-Gonzáles et al. 2005, Alvarado et al. 2008). In Brazil from Ceará, Rio Grande do Norte (Albuquerque 1986), Pernambuco (Tommasi 1970), Alagoas (Miranda et al. 2012), Bahia (Magalhães et al. 2005), Trindade oceanic island off Espírito Santo (Tommasi 1970) and Rio de Janeiro (Manso 1993). Found from 1 to 384m. In this study, recorded for the first time in the State of Paraíba, between 10 and 34 m.

##### Remarks.

Lives in bottoms of coral, dead shells (Tommasi 1970), calcareous algae, in all reef zones, mangroves, seagrass beds, below rocks, in coral colonies, and under sponges (Hendler et al. 1995). The presence of alimentary particles on the arms during the day, when *Ophiocoma wendtii* is hidden inside shelters, suggests that this species is a suspension or detritivorous feeder (Hendler et al. 1995). All samples examined herein have granules on the disk. This differs from the observation of H.L. Clark (1918), who only observed granules in specimens with a disk diameter above 5 mm.

#### Ophiopsila hartmeyeri

Koehler, 1913

http://species-id.net/wiki/Ophiopsila_hartmeyeri

Figure 12a–e

##### Description.

**Figure 12.**
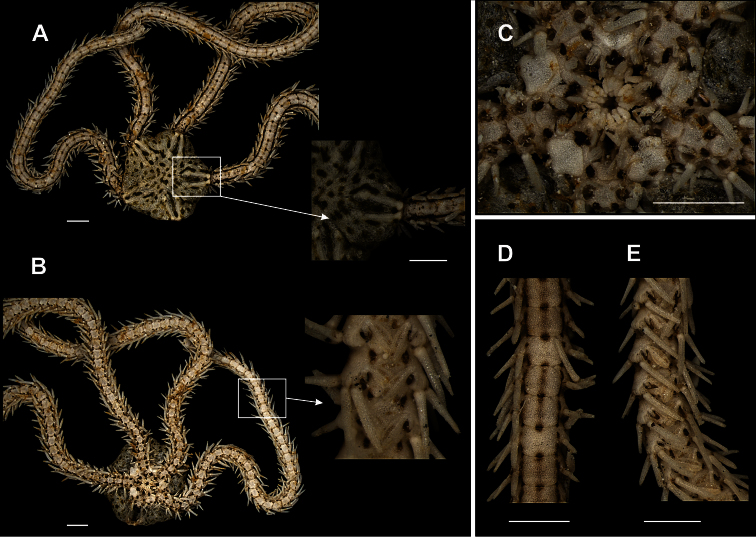
Specimen of the family Ophiocomidae (**a–e**). *Ophiopsila hartmeyeri* **A** dorsal view, detail of the radial shields **B** ventral view, detail of the tentacle scale **C** jaw **D** dorsal view of the arms **E** ventral view of the arms. Scale bar = 1 mm.

Disk circular (dd = 1.30 to 6.90 mm). Covered by imbricating scales of different sizes, largest between radial shields and on interradial margin of disk (Fig. 12a). Radial shields narrow, long, broadly separated, distal end slightly broader, distinct from remaining disk because of their white coloration (Fig. 12a). Numerous olive-green patches on the dorsal and ventral sides of the disk (Fig. 12a). Ventral interradius with scales similar to dorsal disk surface (Fig. 12b). Bursal slits broad and elongated. Oral shields large, diamond-shaped, laterally broadened, some with a dark spot on distal margin. Two spatulate oral papillae (Fig. 12c), borders slightly denteate, on each margin of jaw, the outer one slightly longer. A cluster of dental papillae on apex of jaw (Fig. 12c). Dorsal arm plate slightly wider than long, distal border slightly wider than anterior border (Fig. 12d). Ventral arm plate longer than wide, pentagonal, posterior margin concave (Fig. 12e). Two tentacle scales (Fig. 12b, e), outer smaller and inner longer, flattened and overreaching median ventral plane of arm. Tentacle pore large. Four to six arm spines, ventral one longest and slightly curved (Fig. 12d, e). Remaining spines decrease in size ventralwards, with small denticles at apex. Two close brown stripes on lateral arm plate, and a lighter median band on the dorsal arm plate (Fig. 12a, d). Ventral surface sometimes with two brown stripes close to insertion of spines.

##### Distribution.

Florida Keys, the Mexican Caribbean, the Antilles, Costa Rica, Colombia, Venezuela, and Brazil (Hendler et al. 1995, Alvarado et al. 2008, Borrero-Pérez et al. 2008). In Brazil, from Abrolhos off southern Bahia (Tommasi 1970). From 12 to 161m (Hendler et al. 1995). Recorded 12 and 30 m in present account.

##### Remarks.

Typically, this is a coralline bottom species (Tommasi 1970). Abreu-Pérez et al. (2005) also record the species for sandy substrates and on rocks and corals. We record this species mainly in rhodolites. *Ophiopsila hartmeyeri* showed a mosaic distribution of characters described for *Ophiopsila maculata* (Verrill, 1899), and *Ophiopsila riisei* Lütken, 1859. *Ophiopsila hartmeyeri* was similar to *Ophiopsila maculata* in having black dots on each oral shield, originally diagnosed by Tommasi (1970). Comparing *Ophiopsila hartmeyeri* with *Ophiopsila riisei*, both share these black spots also on the dorsal surface of the disk, a character previously considered diagnostic for *Ophiopsila riisei* (Koehler 1913). The specimens of *Ophiopsila hartmeyeri* from the Paraíba coast have a smaller number of spines (four or six) than that recorded in the literature (eight arm spines). However, their shape is characteristic for *Ophiopsila hartmeyeri* (sword-shaped). In the present study we follow the older classification, in which the genus *Ophiopsila* belong to the family Ophiocomidae, as this genus does not have a pair of infradental papillae (diagnostic character of Amphiuridae), but has tooth papillae (a cluster of short, granule-like apical papillae on the dental plate) (one of the diagnostic characters of Ophiocomidae). Martynov (2010) proposed transfering the genus *Ophiopsila* to the familyAmphiuridade on the basis of the morphology of the lateral arm plate. Murakami (1963), in his exaustive study on the dental and oral plates, suggests that the dental plate of the genus *Ophiopsila* is most closely related to the family Ophionereididae. The systematic position of *Ophiopsila* is thus still uncertain and needs further studies.

### Family Ophiodermatidae Ljungman, 1867

#### Ophioderma appressa

(Say, 1825)

http://species-id.net/wiki/Ophioderma_appressa

Figure 13a–e , 14f

##### Description.

**Figure 13.**
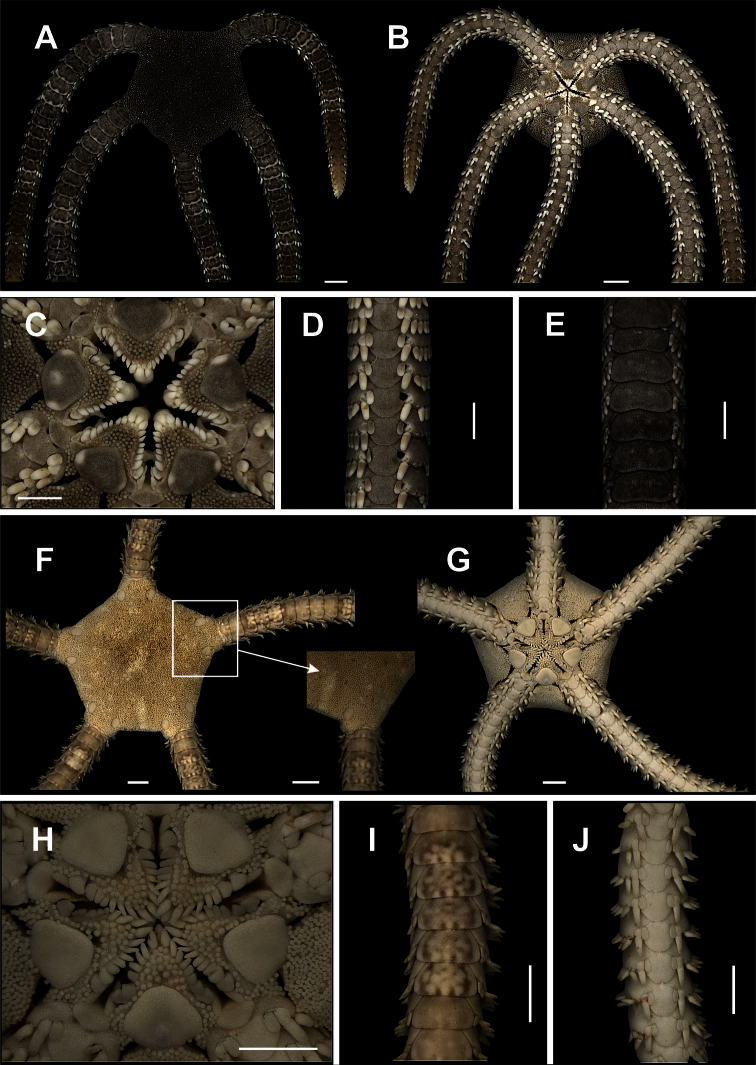
Species of the family Ophiodermatidae (**a–d**). *Ophioderma appressa* **A** dorsal view **B** ventral view **C** jaw **D** ventral view of the arms **E** ventral view of the arms. *Ophioderma cinerea* **F** dorsal view, detail of the radial shields **G** ventral view **H** jaw **I** dorsal view of the arms **J** ventral view of the arms. Scale bar = 1 mm.

Disk circular to pentagonal (dd = 4.18 to 7.89 mm), covered by small granules (Fig. 13a). Radial shields oval and covered with granules (Fig. 13a). Ventral interradius covered by similar granules. Four short bursal slits (Fig. 13b). Oral shields oval (Fig. 13c). Adoral shields broadened laterally, not covered by granules (Fig. 13c). Seven or eight oral papillae on each side of jaw angle (Fig. 13c), the three proximal ones small and elongated the last one narrow and partially covered by the previous papilla. Single apical papillae. Dorsal arm plate longer than wide, with distal margin rounded (Fig. 13e). Seven small, compressed, arm spines, the ventralmost one partially covered by outer tentacle scale. Two tentacle scales, the inner one longest (Fig. 13d).

**Figure 14.**
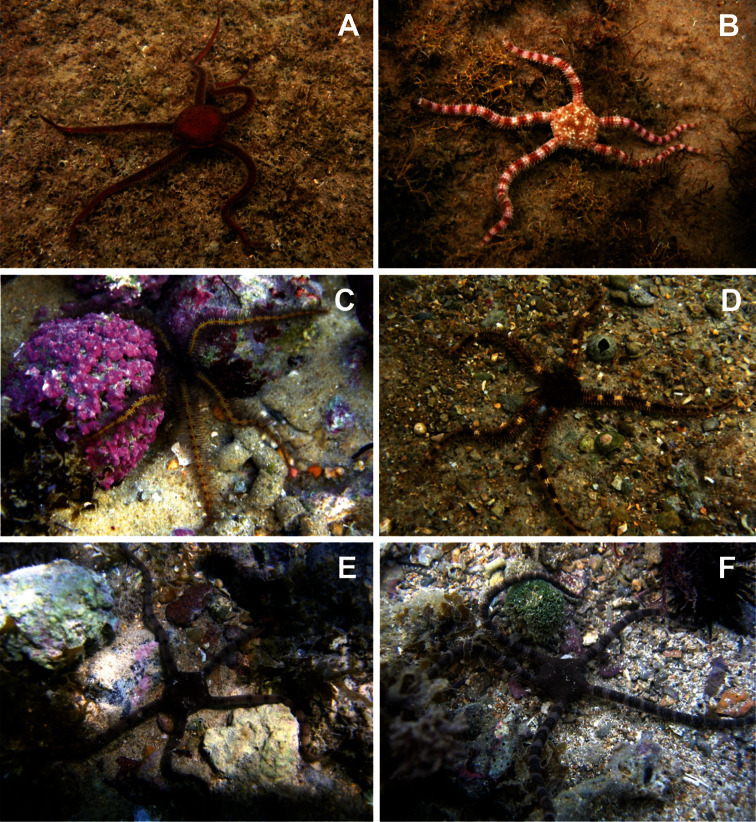
Some ophiurans in their natural habitat. **A** *Ophiomyxa flaccida* **B** *Ophiolepis impressa* **C** *Ophiothrix angulata* **D** *Ophiocoma echinata* **E** *Ophioderma cinerea* **F** *Ophioderma appressa*. Photos by Thelma L. P. Dias.

##### Distribution.

Western Atlantic from Bermuda, South Carolina, the islands off southern Florida, Texas offshore reefs, the Bahamas, the Antilles, Mexican Caribbean, Belize, Honduras, Costa Rica, Panama, islands off Caribbean Colombia, Venezuela, and Brazil (Tommasi 1970, Hendler et al. 1995, Chavarro et al. 2004, Durán-Gonzáles et al. 2005, Alvarado et al. 2008). In Brazil from Paraíba, Pernambuco (Rathbun 1879), Alagoas (Miranda et al. 2012), Bahia (Rathbun 1879), Rio de Janeiro, and São Paulo (Tommasi 1970). Intertidal to 364 m deep. Recorded from 10 to 35 m in the present account.

##### Remarks.

This cryptic species lives in reef environments, seagrass beds, on gravel and coral rubble. Usually found together with other ophiuroids, such as *Ophioderma cinerea*, *Ophiocoma echinata*, and *Ophiocoma wendtii* (Hendler et al. 1995). According to Hendler et al. (1995), records from the East Atlantic are based on misidentified specimens. This species presumably has a vitellaria larva (Hendler 1979b, Hendler and Littman 1986). It is variable both in color and in morphology (Hendler et al. 1995). Among the variable characters are the number of arms pines, that may vary from 7 to 10 spines; Ziesenhenne (1955) observed specimens with 9 to 10 arm spines.

#### Ophioderma cinerea

Müller & Troschel, 1842

http://species-id.net/wiki/Ophioderma_cinerea

Figure 13f–j , 14e

##### Description.

Disk circular to pentagonal (dd = 4.96 to 9.67 mm). Covered by small granules, except on radial shields (Fig. 13f). Radial shields oval. Ventral interradius covered by granules similar to dorsal ones (Fig. 13g). Four short bursal slits. Oral shields heart-shaped (Fig. 13h). Adoral shields small, laterally broadened, not covered by granules. Seven to nine oral papillae on each side of jaw angle (Fig. 13h), the three proximal ones small and elongate, the following ones becoming progressively wider, the last being elongate, narrow and partially covered by preceeding papilla. Single long and robust apical papilla. Dorsal arm plate wider than long (Fig. 13i). Ventral arm plate longer than large, with distal margin rounded (Fig. 13j). Seven to nine small and compressed arm spines, the ventral largest and partially covered by the outer tentacle scale. Two tentacle scales, the inner one long and narrow, the outer one small and subtriangular.

##### Distribution.

The Bahamas, the islands off southern Florida, the Antilles, Mexican Caribbean, Belize, Honduras, Costa Rica, Panama, coast and islands off Caribbean Colombia, Venezuela, and Brazil (H.L. Clark 1915, 1933, Tommasi 1970, Chavarro et al. 2004, Durán-Gonzáles et al. 2005, Alvarado et al. 2008, Hernández-Herrejón et al. 2008). In Brazil from Ceará (Lima-Verde 1969), Rio Grande do Norte (Albuquerque 1986), Paraíba (Gondim et al. 2008), the oceanic island Fernando de Noronha off Pernambuco (Tommasi 1970), Alagoas (Miranda et al. 2012), Abrolhos off Bahia (Tommasi 1970), Bahia (H.L. Clark 1915, Costa and Costa 1962), oceanic island Trindade off Espírito Santo (Brito 1971), Rio de Janeiro (Brito 1962), and São Paulo (Netto et al. 2005). Intertidal to 1.718 m. In present study, recorded from 10 to 34 m.

##### Remarks.

This is one of the most common and largest species in the genus, and differs from other *Ophioderma* from Brazil, such as *Ophioderma appressa* (Say, 1825), and *Ophioderma januarii* Lütken, 1856, by the following characteristics: 1. radial shields within granular covering; 2. dorsal arm plates partitioned. Tommasi (unpublished data) suggested that *Ophioderma besnardi* Tommasi, 1970 represents the young of *Ophioderma cinerea* Müller & Troschel, 1842 before the dorsal plates were divided. More detailed studies are currently being developed to elucidate the taxonomic status of these species. It lives in muddy bottoms, corals (Tommasi 1970), mangroves, and seagrass beds (Hendler et al. 1995).

## Bathymetric distribution

**Figure 15.**
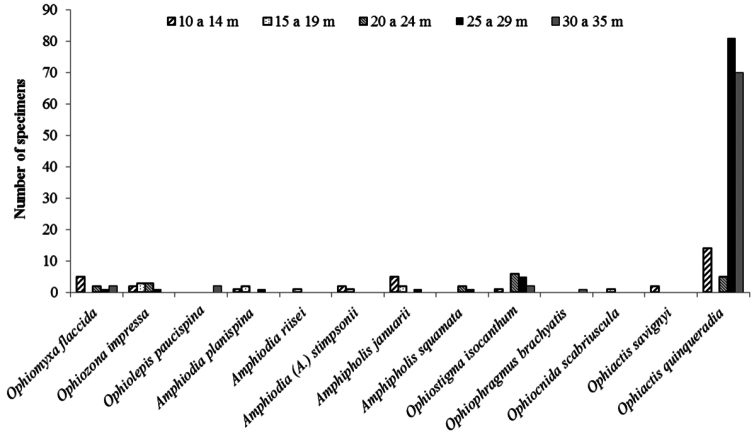
Number of individuals/species of the families Ophiomyxidae, Amphiuridae and Ophiactidae, according to different bathymetric ranges.

**Figure 16.**
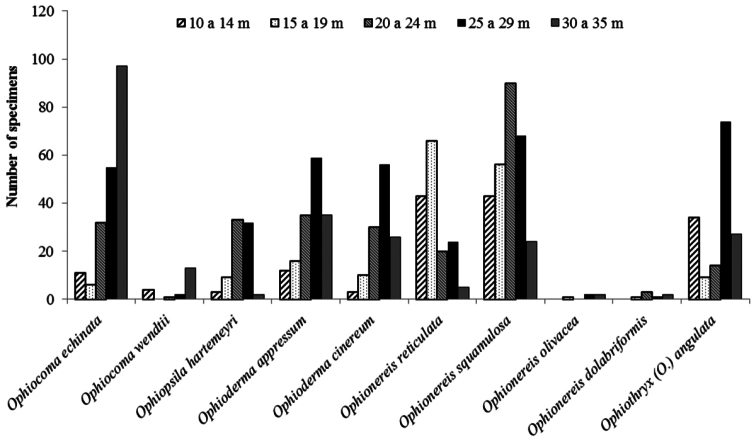
Number of individuals/species of the families Ophiocomidae, Ophiodermatidae, Ophionereididae, Ophiotrichidae and Ophiuridae, according to different bathymetric ranges.

Of the 23 studied species of ophiuroids, 17 occur along practically the whole continental shelf of the State of Paraíba. Only five, *Ophiactis savignyi*, *Ophiophragmus brachyactis*, *Ophiocnida scabriuscula*, *Amphipholis squamata*, and *Amphiodia riisei* had punctual occurrence, being recorded from only one or two collection sites (Fig. 15, Tab. 1, Supplementary material). *Ophiothrix angulata* was the most common, occurring in 73% of the collection stations (Tab. 2, Fig. 16). The bathymetric range of three species, *Ophiolepis impressa*, *Ophiocnida scabriuscula*, and *Ophiocoma echinata* was extended. *Ophionereis squamulosa* was the most abundant species, representing 17.04% of the specimens studied, being more abundant in isobaths 20–24 m deep (Fig. 16). Figure 1 indicates the abundance of specimens in each collection point studied over the continental shelf of Paraíba state, in the isobaths studied.

**Figure 17.**
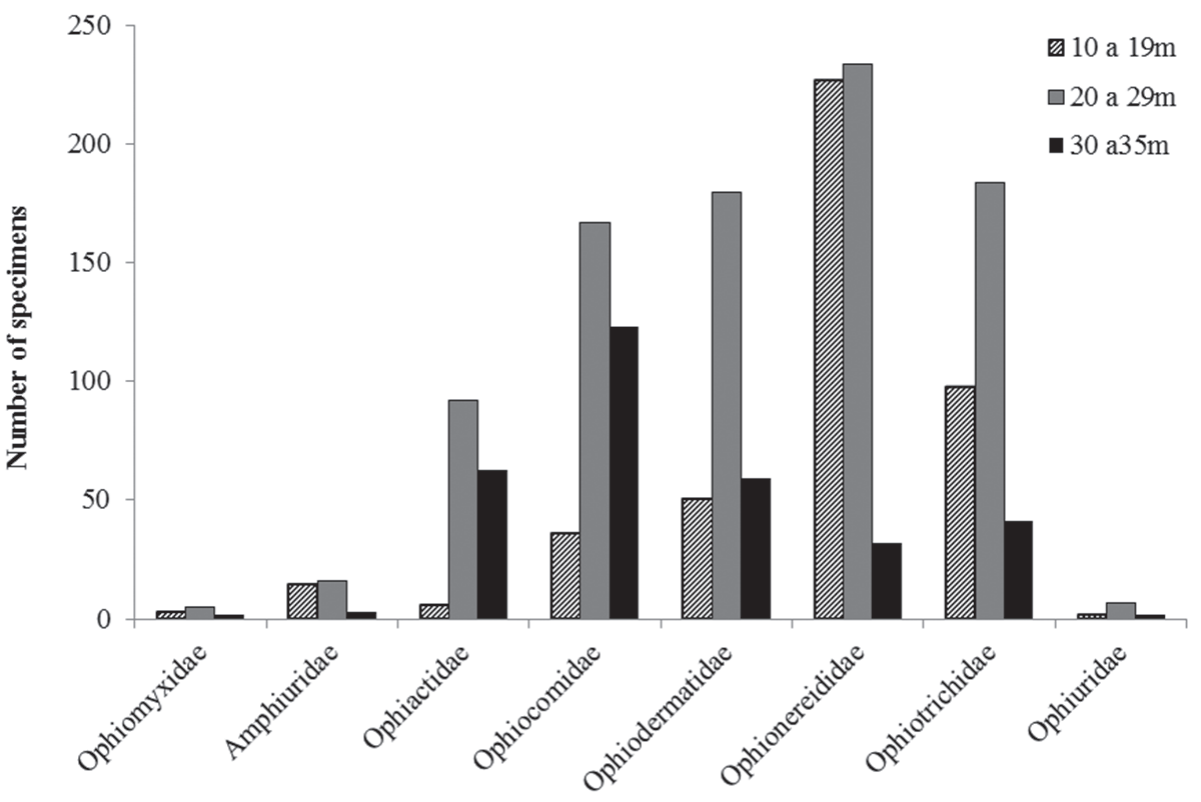
Number of individuals per family, according to different bathymetric ranges.

Among the eight recorded families, Amphiuridae, Ophiactidae, Ophiomyxidae, and Ophiuridae had a patchy occurrence. On the other hand, Ophiocomidae, Ophiodermatidae, Ophionereididae, and Ophiotrichidae were well represented, being frequent over all the extent of the continental shelf (Fig. 17).

In general the species richness was constant along the different depth intervals, the highest species richness occurring between 26 and 30 m (n = 19 spp.) and the lowest species richness occurred between 31 and 35 m (n = 11 spp.) (Fig. 15, 16). Essentially the same pattern was observed for individual families, all families occurred with their greatest numbers at depths between 20 and 29 m (Tab. 2, Supplementary Material) (Fig. 17).

The taxa Ophiactidae and Ophiocomidae showed a similar bathymetric occurrence, being best represented in the intervals 20-29 m and 30–35 m (Fig. 17), and least represented in the interval 10–19 m (Fig. 17). Ophiodermatidae showed the same depth occurrence, but the number of specimens was similar at 10–19 m and 30–35 m (Fig. 16). Ophiomyxidae, Ophionereididae, Ophiotrichidae, and Ophiuridae formed another grouping, in which the interval with the smallest number of specimens was 30–35 m.

## General discussion

The ophiuroid fauna recorded for the continental shelf of the State of Paraíba is composed of species with a large bathymetric distribution, considering that most of them occur from shallow waters to depths greater than 50 m, as pointed out by Tommasi (1970). They are still common along most of the Western Tropical Atlantic, being recorded along the Brazilian coast and in the Caribbean Sea (see Hendler et al. 1995).

We recorded two supposedly cosmopolitan species, *Amphipholis squamata* and *Ophiactis savignyi*, and one amphiatlantic species, *Ophionereis reticulata*. The first two species are visually abundant in the intertidal zone of Paraíba coast, mainly associated with sponges and macroalgae, but were very scarce sampled on the continental shelf. Only two and one specimen of these species was sampled in this study. This may be due to scarcity in the biological material collected, which was composed mainly of calcareous unattached algae (rhodoliths), composed by are calcareous and rigid thallus. Another possibility relates to a possible low abundance of *Ophiactis savignyi* in deeper waters.

The occurrence of the fauna of ophiuroids on the continental platform of the State of Paraíba could be the result of the homogeneity of the bottom types along the sampled area (Coutinho 1996). The availability of food, oxygen and water movement may be sufficient to guarantee a large bathymetric distribution (Fell 1966). Comparing the number of species found in this study (n = 23 spp.) with those recorded for other states in the northeastern region of Brazil, such as the States of Bahia (Magalhães et al. 2005, Manso et al. 2008 – 42 spp.), Ceará (Martins and Martins de Queiroz 2006 – 12 spp.), Pernambuco (Lima and Fernandes 2009 – 15 spp.), Alagoas (Miranda et al. 2012 – 19 spp.), and Sergipe (Manso and Farias 1999 – 3 spp.), we conclude that the species richness found for the continental shelf of the State of Paraíba is representative in comparison with for the total species recorded for the most studied region in Brazil, the southeastern coast. For example, 61 ophiuroid species are known only for the State of São Paulo (Borges et al. 2002, Hadel et al. 1999), where several studies were performed. It is evident that an increase in sampling effort in northeastern Brazilian coast may increase the number of species of this taxon.

In general the most diverse places in the Western Atlantic Ocean with respect to the ophiuran fauna are the Gulf of Mexico (182 spp.), and the Caribbean Sea (148 spp.) (Pawson et al. 2009, Alvarado 2011). On the Brazilian coast a total of 134 species are currently known (Barboza and Borges 2012, Gondim et al. 2012), which is quite similar to the fauna recorded for the Gulf of Mexico and Caribbean.

This suggests that, despite the existence of the barrier of the Amazon River, there is some connectivity between some Caribbean species and those found in the South Atlantic. Studying reef fishes, Joyeux et al. (2001) indicate that an occasional bridge of larval flow can interrupt the isolation between Brazil and the Caribbean. Moreover, according to Collette and Rützler (1977), below 50 m depth, sponges and gorgonians can function as “stepping stones” providing habitat for reef fish species, linking the Caribbean and Brazilian faunas. Although this has not been tested for brittle stars, considering that several species in Brazil and the Caribbean are associated with sponges and gorgonians, this could also be a possibility to explain the similarity in species composition between Brazil and the Caribbean ophiuroids.

Although it has long been postulated that diversity in marine species or communites may follow latitudinal gradients with diversity peaking at the equator and declining towards higher latitudes (Hillebrand 2004, Pianka 1966), the diversity of echinoderms seems to be higher in the southeastern Atlantic, reaching a peak in Antarctica (O’Loughlin et al. 2011). Southern Ocean sites may have acted as refuge, allowing the persistence of crinoid- and ophiuroid-dominated assemblages, which were widespread in the Paleocene, and were apparently displaced by the radiation of mobile predators (Bowden et al. 2011). There are now 129 described species of ophiuroids from the Antarctic (De Broyer and Danis 2011). From a taxonomic point of view even sporadic collecting efforts are extremely important documents of scientific knowledge, and scientific collections represent the main source of knowledge on the biodiversity of a given region (Zaher and Young 2003).

## Acknowledgements

We are grateful to the Federal University of Paraíba, for providing the infrastructure enabling this research, to the National Science Foundation (CNPq) for the scholarships that contributed to this research. MLC is supported by a CNPq productivity research grant (Process number: 300198/2010-8). Special thanks are due to our Graduate Course (Programa de Pós-Graduação em Ciências Biológicas) through Project CT-Infra and Projeto Biota - Paraíba: Macrofauna de Praias com Substrato Consolidado da Zona de Entre-Marés ao Infralitoral, financed by Conselho Nacional de Desenvolvimento Científico e Tecnológico - CNPq, through Edital Universal (Process n° 484601/2007-5), with which the essential photographic equipment for the illustration of specimens was obtained. We also wish to express our sincere gratitude to two anonymous reviewers for their critical reading of the manuscript and constructive comments, and Carolina Nunes Liberal for help with photographs.

## Supplementary Material

XML Treatment for Ophiomyxa flaccida

XML Treatment for Ophiolepis impressa

XML Treatment for Ophiolepis paucispina

XML Treatment for Amphiodia planispina

XML Treatment for Amphiodia riisei

XML Treatment for Amphipholis januarii

XML Treatment for Amphipholis squamata

XML Treatment for Amphiura stimpsoni

XML Treatment for Ophiocnida scabriuscula

XML Treatment for Ophiophragmus brachyactis

XML Treatment for Ophiostigma isocanthum

XML Treatment for Ophiothrix (Ophiothrix) angulata

XML Treatment for Ophiactis quinqueradia

XML Treatment for Ophiactis savignyi

XML Treatment for Ophionereis reticulata

XML Treatment for Ophionereis squamulosa

XML Treatment for Ophionereis dolabriformis

XML Treatment for Ophionereis olivacea

XML Treatment for Ophiocoma echinata

XML Treatment for Ophiocoma wendtii

XML Treatment for Ophiopsila hartmeyeri

XML Treatment for Ophioderma appressa

XML Treatment for Ophioderma cinerea

## Appendix I

Collection data of voucher specimens (spec.) collected at Paraíba State continental shelf, Northeastern Brazil. (doi: 10.3897/zookeys.307.4673.app1) File format: Mircrosoft Word Document (doc).

## Appendix II

Data on the size (measured by disk diameter), number of individuals and relative abundance of voucher specimens collected at Paraíba State continental shelf, Northeastern Brazil. SD = standard deviation. (doi: 10.3897/zookeys.307.4673.app2) File format: Mircrosoft Word Document (doc).
