# Supplementary material for: A taxonomic guide to the brittle-stars (Echinodermata, Ophiuroidea) from the State of Paraíba continental shelf, Northeastern Brazil
Source: Zookeys. 2013 Jun 10;(307):45–96. doi: 10.3897/zookeys.307.4673 (PMC3689063; doi:10.3897/zookeys.307.4673)
Supplement: Supplementary file 24 — Collection data of voucher specimens (spec.) collected at Paraíba State continental shelf, Northeastern Brazil. (doi: 10.3897/zookeys.307.4673.app1) File format: Mircrosoft Word Document (doc). [file ZooKeys-307-045-s001.doc]

**TABLE 1.** Collection data of voucher specimens (spec.) collected at Paraíba State continental shelf, Northeastern Brazil.

| **Station** | **Coordinates** | **Depth (m)** | **Taxon/Voucher/ Nº specimens** | **Total specimens number** |
| --- | --- | --- | --- | --- |
| 01 | 7°34' S; 34°45' W | 11 | *O. flaccida* (UFPB.ECH.393, 1 spec.), *O. angulata* (UFPB.ECH.788, 1 spec.), *O. quinqueradia* (UFPB.ECH.423, 6 spec.), *O. reticulata* (UFPB.ECH.640, 4 spec.), *O. echinata* (UFPB.ECH.406, 4 spec.) | 16 |
| 02 | 7°34 'S; 34°42' W | 20 | *O. reticulata* (UFPB.ECH.327, 1 spec.), *O. squamulosa* (UFPB.ECH.898, 2 spec.), *O. hartmeyeri* (UFPB.ECH.392, 11 spec.), *O. cinerea* (UFPB.ECH.420, 2 spec.) | 16 |
| 03 | 7°34' S; 34°39' W | 26 | *O. angulata* (UFPB.ECH.790, 1 spec.), *O. squamulosa* (UFPB.ECH.656, 14 spec.), *O. hatmeyeri* (UFPB.ECH.767, 2 spec.), *O. cinerea* (UFPB.ECH.996, 1 spec.) | 18 |
| 04 | 7°34' S; 34°36' W | 33 | *O. flaccida* (UFPB.ECH.442, 1 spec.), *O. angulata* (UFPB.ECH.403, 3 spec.), *O. quinqueradia* (UFPB.ECH.261, 15 spec.), *O. reticulata* (UFPB.ECH.1080, 1 spec.), *O. echinata* (UFPB.ECH.260, 26 spec.), *O. wendtii* (UFPB.ECH.549, 2 spec.), *O. appressa* (UFPB.ECH.485, 1 spec.) | 49 |
| 05 | 7°31' S; 34'31' W | 34 | *O. angulata* (UFPB.ECH.541, 2 spec.), *O. quinqueradia* (UFPB.ECH.425, 13 spec.), *O. echinata* (UFPB.ECH.370, 11 spec.), *O. wendtii* (UFPB.ECH.451, 1 spec.), *O. cinerea* (UFPB.ECH.751, 1 spec.) | 28 |
| 06 | 7°31' S; 34°39' W | 24 | *O. angulata* (UFPB.ECH.600, 1 spec.), *O. squamulosa* (UFPB.ECH.636, 4 spec.), *O. hatmeyeri* (UFPB.ECH.426, 8 spec.), *O. apressa* (UFPB.ECH.511, 2 spec.), *O. cinerea* (UFPB.ECH.595, 3 spec.) | 18 |
| 07 | 7°31' S; 34°42' W | 24 | *O. squamulosa* (UFPB.ECH.891, 1 spec.), *O. hartmeyeri* (UFPB.ECH.938, 2 spec.) | 3 |
| 10 | 7°28' S; 34°40' W | 14 | *O. squamulosa* (UFPB.ECH.1084, 1 spec.), *O.dolabriformis* (UFPB.ECH.1082, 1 spec.), *O. hartmeyeri* (UFPB.ECH.1099, 1 spec.) | 3 |
| 11 | 7°28' S; 34°37 'W | 24 | *O. squamulosa* (UFPB.ECH.896, 4 spec.), *O. dolabriformis* (UFPB.ECH.1044, 1 spec.), *O. hartmeyeri* (UFPB.ECH.1027, 1 spec.) | 6 |
| 12 | 7°28' S; 34°34' W | 30 | *O. isocanthum* (UFPB.ECH.533, 1 spec.), *O. angulata* (UFPB.ECH.515, 1 spec.), *O. reticulata* (UFPB.ECH.480, 2 spec.), *O. squamulosa* (UFPB.ECH.844, 1 spec.), *O. dolabriformis* (UFPB.ECH.748, 1 spec.), *O. olivacea* (UFPB.ECH.910, 2 spec.), *O. echinata* (UFPB.ECH.507, 3 spec.), *O. wendtii* (UFPB/ECH.630, 6 spec.), *O. appressa* (UFPB.ECH.362, 5 spec.), *O. cinerea* (UFPB.ECH.361, 2 spec.) | 24 |
| 13 | 7°25' S; 34°34' W | 30 | *O. angulata* (UFPB.ECH.552, 1 spec.), *O. squamulosa* (UFPB.ECH.1057, 1 spec.), *O. wendtii* (UFPB.ECH.467, 9 spec.), *O. appressa* (UFPB.ECH.309, 7 spec.) | 18 |
| 14 | 7°25' S; 34°37' W | 20 | *O. angulata* (UFPB.ECH.538, 2 spec.), *O. reticulata* (UFPB.ECH.919, 2 spec.), *O. squamulosa* (UFPB.ECH.644, 9 spec.), *O. hartmeyeri* (UFPB.ECH.466, 4 spec.), *O. appressa* (UFPB.ECH.464, 6 spec.) | 23 |
| 16 | 7°25' S; 34°43' W | 10 | *A. januarii* (UFPB.ECH.496, 1 spec.), *A. stimpsoni* (UFPB.ECH.366, 1 spec.), *O. reticulata* (UFPB.ECH.925, 1 spec.), *O. hartmeyeri* (UFPB.ECH.468, 1 spec.), *O. angulata* (UFPB.ECH.537, 2 spec.), *O. echinata* (UFPB.ECH.461, 3 spec.), *O. cinerea* (UFPB.ECH.363, 1 spec.) | 10 |
| 17 | 7°22' S; 34°44' W | 10 | *A. januarii* (UFPB.ECH.576, 1 spec.), *O. reticulata* (UFPB.ECH.559, 3 spec.), *O. squamulosa* (UFPB.ECH.584, 1 spec.) | 5 |
| 18 | 7°18' S; 34°36' W | 14 | *A. januarii* (UFPB.ECH.471, 1 spec.), *O. reticulata* (UFPB.ECH.475, 2 spec.), *O. echinata* (UFPB.ECH.493, 1 spec.) | 4 |
| 20 | 7°21'S; 34°38' W | 30 | *O. paucispina* (UFPB.ECH.562, 1 spec.), *O. angulata* (UFPB.ECH.590, 4 spec.), *O. quinqueradia* (UFPB.ECH.1091, 4 spec.), *O. reticulata* (UFPB.ECH.638, 2 spec.), *O. dolabriformis* (UFPB.ECH.749, 1 spec.), *O. echinata* (UFPB.ECH.588, 2 spec.), *O. appressa* (UFPB.ECH.583, 3 spec.) | 17 |
| 21 | 7°18' S; 34°33' W | 30 | *O. quinqueradia* (UFPB.ECH.300, 10 spec.), *O. echinata* (UFPB.ECH.606, 1 spec.), *O. cinerea* (UFPB.ECH.613, 1 spec.) | 12 |
| 22 | 7°21' S; 34°41' W | 28 | *O. angulata* (UFPB.ECH.738, 1 spec.), *O. appressa* (UFPB.ECH.817, 1 spec.) | 2 |
| 23 | 7°18' S; 34°00' W | 18 | *O. hartmeyeri* (UFPB.ECH.304, 1 spec.) | 1 |
| 27 | 7°15'05" S; 34°42' W | 16 | *O. reticulata* (UFPB.ECH.916, 1 spec.), *O. squamulosa* (UFPB.ECH.907, 2 spec.), *O. hartmeyeri* (UFPB.ECH.937, 1 spec.), *O. appressa* (UFPB.ECH.678, 1 spec.) | 5 |
| 29 | 7°15'5" S; 34°36' W | 28 | *O. impressa* (UFPB.ECH.491, 1 spec.), *O. isocanthum* (UFPB.ECH.1086, 1 spec.), *O. angulata* (UFPB.ECH.783, 2 spec.), *O. squamulosa* (UFPB.ECH.841, 2 spec.), *O. hartmeyeri* (UFPB.ECH.1097, 2 spec.), *O. appressa* (UFPB.ECH.614, 8 spec.), *O. cinerea* (UFPB.ECH.750, 9 spec.) | 25 |
| 30 | 7°15'5" S; 34°33' W | 35 | *O. appressa* (UFPB.ECH.814, 1 spec.) | 1 |
| 31 | 7°12'5” S; 34°36' W | 26 | *O. isocanthum* (UFPB.ECH.410, 2 spec.), *O. angulata* (UFPB.ECH.402, 5 spec.), *O. reticulata* (UFPB.ECH.1077, 1 spec.), *O. squamulosa* (UFPB.ECH.843, 8 spec.), *O. olivacea* (UFPB.ECH.909, 2 spec.), *O. echinata* (UFPB.ECH.266, 2 spec.), *O. hartmeyeri* (UFPB.ECH.417, 4 spec.), *O. appressa* (UFPB.ECH.827, 1 spec.), *O. cinerea* (UFPB.ECH.414, 1 spec.) | 28 |
| 32 | 7°12'5" S; 34°39' W | 20 | *O. angulata* (UFPB.ECH.789, 1 spec.), *O. squamulosa* (UFPB.ECH.340, 15 spec.), *O. echinata* (UFPB.ECH.1413, 1 spec.), *O. wendtii* (UFPB.ECH.690, 1 spec.), *O. hartmeyeri* (UFPB.ECH.456, 3 spec.), *O. apressa* (UFPB.ECH.692, 7 spec.), *O. cinerea* (UFPB.ECH.753, 6 spec.) | 34 |
| 33 | 7°13' S; 34°42' W | 20 | *O. angulata* (UFPB.ECH.535, 4 spec.), *O. reticulata* (UFPB.ECH.654, 2 spec.), *O. squamulosa* (UFPB.ECH.747, 16 spec.), *O. echinata* (UFPB.ECH.463, 2 spec.), *O. hartmeyeri* (UFPB.ECH.771, 2 spec.) | 26 |
| 34 | 7°13' S; 34°45' W | 10 | *O. reticulata* (UFPB.ECH.342, 1 spec.) | 1 |
| 35 | 7°10' S; 34°45' W | 27 | *A. planispina* (UFPB.ECH.685, 1 spec.), *A. januarii* (UFPB.ECH.684, 1 spec.), *O. angulata* (UFPB.ECH.514, 3 spec.), *O. reticulata* (UFPB.ECH.484, 4 spec.), *O. echinata* (UFPB.ECH.527, 3 spec.) | 12 |
| 36 | 7°10' S; 34°42' W | 10 | *A. stimpsoni* (UFPB.ECH.990, 1 spec.), *O. angulata* (UFPB.ECH.536, 5 spec.), *O. reticulata* (UFPB.ECH.526, 1 spec.), *O. squamulosa* (UFPB.ECH.517, 6 spec.), *O. hatmeyeri* (UFPB.ECH.531, 1 spec.), *O. appressa* (UFPB.ECH.1001, 1 spec.) | 15 |
| 37 | 7°10' S; 34°38' W | 25 | *O. angulata* (UFPB.ECH.555, 3 spec.), *O. reticulata* (UFPB.ECH.660, 5 spec.), *O. squamulosa* (UFPB.ECH.528, 6 spec.), *O. hartmeyeri* (UFPB.ECH.323, 6 spec.), *O. appressa* (UFPB.ECH.328, 4 spec.), *O. cinerea* (UFPB.ECH.752, 1 spec.) | 25 |
| 38 | 7°37' S; 34°37' W | 27 | *O. cinerea* (UFPB.ECH.580, 2 spec.) | 2 |
| 39 | 7°07' S; 34°40' W | 27 | *O. isocanthum* (UFPB.ECH.1164, 1 spec.), *O. angulata* (UFPB.ECH.565, 5 spec.), *O. reticulata* (UFPB.ECH.646, 4 spec.), *O. squamulosa* (UFPB.ECH.649, 2 spec.), *O. hartmeyeri* (UFPB.ECH.802, 7 spec.), *O. appressa* (UFPB.ECH.587, 1 spec.), *O. cinerea* (UFPB.ECH.121, 3 spec.) | 23 |
| 40 | 7°07' S; 34°43' W | 17 | *O. reticulata* (UFPB.ECH.917, 1 spec.), *O. squamulosa* (UFPB.ECH.885, 1 spec.), *O. hartmeyeri* (UFPB.ECH.429, 1 spec.) | 3 |
| 41 | 7°07' S; 34°46' W | 10 | *O. impressa* (UFPB.ECH.497, 1 spec.), *A. januarii* (UFPB.ECH.545, 1 spec.), *O. reticulata* (UFPB.ECH.513, 7 spec.), *O. squamulosa* (UFPB.ECH.901, 1 spec.) | 10 |
| 42 | 7°07' S; 34°47' W | 10 | *A. januarii* (UFPB.ECH.993, 1 spec.), *O. angulata* (UFPB.ECH.786, 1 spec.), *O. savignyi* (UFPB.ECH.428, 2 spec.), *O. reticulata* (UFPB.ECH.742, 4 spec.), *O. squamulosa* (UFPB.ECH.668, 1 spec.) | 9 |
| 43 | 7°04' S; 34°44' W | 16 | *A. riisei* (UFPB.ECH.991, 1 spec.), *O. angulata* (UFPB.ECH.360, 4 spec.), *O. reticulata* (UFPB.ECH.557, 2 spec.), *O. appressa* (UFPB.ECH.592, 1 spec.), *O. cinerea* (UFPB.ECH.347, 1 spec.) | 9 |
| 44 | 7°04' S; 34°41' W | 22 | *O. angulata* (UFPB.ECH.780, 1 spec.), *O. squamulosa* (UFPB.ECH.745, 4 spec.), *O. dolabriformis* (UFPB.ECH.1045, 1 spec.), *O. appressa* (UFPB.ECH.341, 2 spec.) | 8 |
| 45 | 7°04' S; 34°38' W | 26 | *O. isocanthum* (UFPB.ECH.415, 1 spec.), *O. angulata* (UFPB.ECH.421, 9 spec.), *O. squamulosa* (UFPB.ECH.883, 4 spec.), *O. hartmeyeri* (UFPB.ECH.407, 3 spec.), *O. appressa* (UFPB.ECH.453, 9 spec.), *O. cinerea* (UFPB.ECH.412, 8 spec.) | 34 |
| 46 | 7°04' S; 34°36' W | 34 | *O. isocanthum* (UFPB.ECH.321, 1 spec.), *O. squamulosa* (UFPB.ECH.637, 1 spec.), *O. appressa* (UFPB.ECH.131, 9 spec.), *O. cinerea* (UFPB.ECH.135, 8 spec.) | 19 |
| 47 | 7°01' S; 34°30' W | 26 | *O. angulata* (UFPB.ECH.345, 10 spec.), *O. squamulosa* (UFPB.ECH.625, 10 spec.), *O. hatmeyeri* (UFPB.ECH.408, 5 spec.), *O. appressa* (UFPB.ECH.457, 6 spec.), *O. cinerea* (UFPB.ECH.346, 8 spec.) | 39 |
| 48 | 7°01' S; 34°41' W | 24 | *O. appressa* (UFPB.ECH.505, 9 spec.) | 9 |
| 50 | 7°01' S; 34°47'05" W | 11 | *A. planispina* (UFPB.ECH.1033, 1 spec.), *O. reticulata* (UFPB.ECH.1047, 1 spec.) | 2 |
| 53 | 6°57' S; 34°41' W | 26 | *O. squamulosa* (UFPB.ECH.1052, 2 spec.), *O. dolabriformis* (UFPB.ECH.1046, 1 spec.), *O. appressa* (UFPB.ECH.825, 4 spec.) | 7 |
| 54 | 6°57' S; 34°38' W | 30 | *O. brachyactis* (UFPB.ECH.1121, 1 spec.), *O. angulata* (UFPB.ECH.506, 6 spec.), *O. angulata* (UFPB.ECH.540, 5 spec.), *O. squamulosa* (UFPB.ECH.524, 13 spec.), *O. wendtii* (UFPB.ECH.508, 4 spec.), *O. appressa* (UFPB.ECH.812, 5 spec.), *O. cinerea* (UFPB.ECH.525, 9 spec.) | 43 |
| 55 | 6°55' S; 34°40' W | 28 | *O. angulata* (UFPB.ECH.791, 2spec.), *O. squamulosa* (UFPB.ECH.686, 8spec.), *O. wendtii* (UFPB.ECH.689, 1spec.), *O. cinerea* (UFPB.ECH.136, 9spec.) | 20 |
| 56 | 6°55' S; 34°43'05" W | 21 | *A. squamata* (UFPB.ECH.326, 1 spec.), *O. isocanthum* (UFPB.ECH.995, 4 spec.), *O. quinqueradia* (UFPB.ECH.1022, 2 spec.), *O. reticulata* (UFPB.ECH.308, 3 spec.), *O. squamulosa* (UFPB.ECH.658, 24 spec.), *O. dolabriformis* (UFPB.ECH.912, 1 spec.), *O. hatmeyeri* (UFPB.ECH.325, 3 spec.), *O. appressa* (UFPB.ECH.458, 3 spec.), *O. cinerea* (UFPB.ECH.631, 1 spec.) | 42 |
| 57 | 6°55' S; 34°46'05" W | 18 | *O. squamulosa* (UFPB.ECH.884, 5 spec.), *O. hartmeyeri* (UFPB.ECH.933, 1 spec.) | 6 |
| 58 | 6°55' S; 34°38' W | 28 | *O. angulata* (UFPB.ECH.404, 11 spec.), *O. squamulosa* (UFPB.ECH.849, 4 spec.), *O. wendtii* (UFPB.ECH.397, 1 spec.), *O. appressa* (UFPB.ECH.499, 8 spec.) | 24 |
| 60 | 6°52' S; 34°46' W | 18 | *A. planispina* (UFPB.ECH.992, 1 spec.), *O. scabriuscula* (UFPB.ECH.994, 1 spec.), *O. reticulata* (UFPB.ECH.921, 10 spec.), *O. squamulosa* (UFPB.ECH.642, 8 spec.), *O. dolabriformis* (UFPB.ECH.913, 1 spec.), *O. olivacea* (UFPB.ECH.911, 1 spec.), *O. echinata* (UFPB.ECH.529, 1 spec.), *O. appressa* (UFPB.ECH.674, 4 spec.) | 27 |
| 61 | 6°52' S; 34º42' W | 20 | *O. flaccida* (UFPB.ECH.455, 1 spec.), *O. impressa* (UFPB/ECH.490, 1 spec.), *O. isocanthum* (UFPB.ECH.567, 1 spec.), *O. angulata* (UFPB.ECH.566, 2 spec.), *O. quinqueradia* (UFPB.ECH.534, 2 spec.), *O. squamulosa* (UFPB.ECH.481, 8 spec.), *O. echinata* (UFPB.ECH.440, 16 spec.), *O. cinerea* (UFPB.ECH.503, 8 spec.) | 39 |
| 62 | 6°52' S; 34°40' W | 32 | *O. angulata* (UFPB.ECH.539, 10 spec.), *O. appressa* (UFPB.ECH.530, 2 spec.) | 12 |
| 63 | 6°50' S; 34°42' W | 30 | *O. flaccida* (UFPB.ECH.400, 1 ex), *O. quinqueradia* (UFPB.ECH.617, 2 spec.), *O. squamulosa* (UFPB.ECH.1053, 1 spec.), *O. appressa* (UFPB.ECH.819, 2 spec.), *O. cinerea* (UFPB.ECH.577, 3 spec.) | 9 |
| 64 | 6°50' S; 34°44' W | 26 | *A. squamata* (UFPB.ECH.343, 1 spec.), *O. angulata* (UFPB.ECH.516, 11spec.), *O. quinqueradia* (UFPB.ECH.478, 4 spec.), *O. reticulata* (UFPB.ECH.344, 10 spec.), *O. squamulosa* (UFPB.ECH.320, 4 spec.), *O. hartmeyeri* (UFPB.ECH.804, 1 spec.), *O. appressa* (UFPB.ECH.675, 6 spec.), *O. cinerea* (UFPB.ECH.479, 1 spec.) | 38 |
| 65 | 6º 50' S; 34º47' W | 18 | *O. impressa* (UFPB.ECH.657, 2 spec.), *A. planispina* (UFPB.ECH.1090, 1 spec.), *A. januarii* (UFPB.ECH.662, 1 spec.), *A. stimpsoni* (UFPB.ECH.774, 1 spec.), *O. angulata* (UFPB.ECH.624, 3 spec.), *O. reticulata* (UFPB.ECH.681, 40 spec.), *O. squamulosa* (UFPB.ECH.680, 18 spec.), *O. echinata* (UFPB.ECH.602, 4 spec.), *O. hatmeyeri* (UFPB.ECH.633, 4 spec.) | 74 |
| 66 | 6°50' S; 34°50' W | 10 | *O. angulata* (UFPB.ECH.575, 9 spec.), *O. squamulosa* (UFPB.ECH.564, 20 spec.), *O. echinata* (UFPB.ECH.929, 1 spec.), *O. appressa* (UFPB.ECH.1354, 1 spec.) | 31 |
| 67 | 6°46' S; 34°53' W | 10 | *O. reticulata* (UFPB.ECH.920, 3 spec.) | 3 |
| 68 | 6°46' S; 34°50' W | 14 | *O. flaccida* (UFPB.ECH.449, 2spec.), *O. reticulata* (UFPB.ECH.512, 12 spec.), *O. wendtii* (UFPB.ECH.672, 2 spec.), *O. appressa* (UFPB.ECH.998, 1 spec.), *O. cinerea* (UFPB.ECH.834, 2 spec.) | 19 |
| 69 | 6°46' S; 34°47' W | 18 | *O. appressa* (UFPB.ECH.1004, 2 spec.) | 2 |
| 71 | 6°43' S; 34°51' W | 22 | *O. appressa* (UFPB.ECH.811, 2 spec.) | 2 |
| 74 | 6°43' S; 34°54' W | 10 | *O. reticulata* (UFPB.ECH.329, 1 spec.) | 1 |
| 77 | 6°39'05" S; 34°49' W | 20 | *O. isocanthum* (UFPB.ECH.1087, 1spec.), *O. appressa* (UFPB.ECH.837, 1spec.) | 2 |
| 79 | 6°39' S; 34°43' W | 22 | *O. appressa* (UFPB.ECH.813, 3 spec.) | 3 |
| 80 | 6°37' S; 34°51' W | 20 | *O. angulata* (UFPB.ECH.364, 3 spec.), *O. quinqueradia* (UFPB.ECH.683, 1 spec.), *O. reticulata* (UFPB.ECH.305, 12 spec.), *O. echinata* (UFPB.ECH.605, 1 spec.), *O. appressa* (UFPB.ECH.365, 11 spec.), *O. cinerea* (UFPB.ECH.754, 5 spec.) | 33 |
| 81 | 6°37' S; 34°54' W | 12 | *O. flaccida* (UFPB.ECH.448, 2 spec.), *O. impressa* (UFPB/ECH.486, 1 spec.), *O. angulata* (UFPB.ECH.259, 14 spec.), *O. quinqueradia* (UFPB.ECH.289, 6 spec.), *O. reticulata* (UFPB.ECH.477, 1 spec.), *O. squamulosa* (UFPB.ECH.687, 6 spec.), *O. echinata* (UFPB.ECH.257, 11 spec.), *O. wendtii* (UFPB.ECH.554, 2 spec.), *O. appressa* (UFPB.ECH.579, 1 spec.) | 44 |
| 83 | 6°33' S; 34°57' W | 12 | *O. angulata* (UFPB.ECH.424, 1 spec.), *O. appressa* (UFPB.ECH.826, 1 spec.) | 2 |
| 84 | 6°33' S; 34°54' W | 14 | *O. isocanthum* (UFPB.ECH.1163, 1 spec.), *O. angulata* (UFPB.ECH.733, 3 spec.) | 4 |
| 85 | 6°33' S; 34°51' W | 20 | *O. flaccida* (UFPB.ECH.498, 1 spec.), *O. squamulosa* (UFPB.ECH.655, 3 spec.), *O. appressa* (UFPB.ECH.501, 2 spec.), *O. cinerea* (UFPB.ECH.695, 4 spec.) | 10 |
| 86 | 6°33' S; 34°47' W | 26 | *O. flaccida* (UFPB.ECH.450, 1 spec.), *O. impressa* (UFPB/ECH.488, 2 spec.), *O. angulata* (UFPB.ECH.373, 5 spec.), *O. quinqueradia* (UFPB.ECH.288, 77 spec.), *O. squamulosa* (UFPB.ECH.547, 1 spec.), *O. echinata* (UFPB.ECH.338, 47 spec.), *O. appressa* (UFPB.ECH.829, 6 spec.), *O. cinerea* (UFPB.ECH.454, 2 spec.) | 141 |
| 87 | 6°29' S; 34°48' W | 30 | *O. quinqueradia* (UFPB.ECH.286, 26 spec.), *O. squamulosa* (UFPB.ECH.696, 7 spec.), *O. echinata* (UFPB.ECH.255, 56 spec.), *O. hartmeyeri* (UFPB.ECH.770, 2 spec.), *O. appressa* (UFPB.ECH.693, 1 spec.), *O. cinerea* (UFPB.ECH.254, 2 spec.) | 95 |
| 88 | 6°29' S; 34°51' W | 22 | *O. cinerea* (UFPB.ECH.258, 1 spec.) | 1 |
| 89 | 6°29' S; 34°54' W | 17 | *O. angulata* (UFPB.ECH.546, 1 spec.), *O. reticulata* (UFPB.ECH.465, 8 spec.), *O. squamulosa* (UFPB.ECH.469, 22 spec.), *O. appressa* (UFPB.ECH.306, 4 spec.), *O. cinerea* (UFPB.ECH.551, 6 spec.) | 40 |
| 90 | 6°29' S; 34°57' W | 12 | *O. squamulosa* (UFPB.ECH.890, 1 spec.) | 1 |
| 91 | 6°26' S; 34°58' W | 14 | *O. squamulosa* (UFPB.ECH.851, 1 spec.), *O. appressa* (UFPB.ECH.820, 7 spec.) | 8 |
| 92 | 6°26' S; 34°55' W | 16 | *O. reticulata* (UFPB.ECH.647, 5 spec.), *O. squamulosa* (UFPB.ECH.650, 8 spec.), *O. hartmeyeri* (UFPB.ECH.803, 1 spec.), *O. appressa* (UFPB.ECH.757, 4 spec.), *O. cinerea* (UFPB.ECH.756, 3 spec.) | 18 |
| 93 | 6°26' S; 34°52' W | 26 | *O. impressa* (UFPB.ECH.127, 2 spec.), *O. angulata* (UFPB.ECH.792, 6 spec.), *O. squamulosa* (UFPB.ECH.846, 3 spec.), *O. echinata* (UFPB.ECH.256, 2 spec.), *O. hartmeyeri* (UFPB.ECH.932, 1 spec.), *O. apressa* (UFPB.ECH.126, 5 spec.), *O. cinerea* (UFPB.ECH.282, 11 spec.) | 30 |
