# Supplementary material for: A taxonomic guide to the brittle-stars (Echinodermata, Ophiuroidea) from the State of Paraíba continental shelf, Northeastern Brazil
Source: Zookeys. 2013 Jun 10;(307):45–96. doi: 10.3897/zookeys.307.4673 (PMC3689063; doi:10.3897/zookeys.307.4673)
Supplement: Supplementary file 25 — Data on the size (measured by disk diameter), number of individuals and relative abundance of voucher specimens collected at Paraíba State continental shelf, Northeastern Brazil. SD = standard deviation. (doi: 10.3897/zookeys.307.4673.app2) File format: Mircrosoft Word Document (doc). [file ZooKeys-307-045-s002.doc]

| **Species** | **Disk diameter** | | **Number of specimens** | **Relative abundance (%)** | | |
| --- | --- | --- | --- | --- | --- | --- |
|  | **Range (mm)** | **Mean (±SD)** |  | **10 to 20m** | **21 to 35m** | **Total** |
| *Ophiomyxa flaccida* (Say, 1825) | 5.67 - 12.16 | 4.00  (**±** 2.16) | 10 | 1.28 | 0.36 | 0.72 |
| *Ophiolepis impressa* Lütken, 1859 | 4.26 - 9.82 | 5.50  (**±** 3.02) | 8 | 0.54 | 0.60 | 0.58 |
| *Ophiolepis paucispina* (Say, 1825) | 2.74 - 3.55 | 2.00  (**±**1.00) | 2 | 0 | 0.24 | 0.14 |
| *Amphiodia planispina* (von Martens, 1867) | 4.46 - 5.80 | 1.50  (**±**0.70) | 4 | 0.54 | 0.12 | 0.29 |
| *Amphiodia riisei* (Lütken, 1859) | 4.16 | - | 1 | 0.18 | 0 | 0.07 |
| *Amphipholis januarii* Ljungman, 1866 | 1.90 - 2.70 | 2.00  (**±**1.00) | 9 | 1.28 | 0.24 | 0.65 |
| *Amphipholis squamata* (Delle Chiaje, 1828) | 1.08 - 2.47 | 2.00  (**±**1.00) | 3 | 0 | 0.36 | 0.21 |
| *Amphiura stimpsoni* Lütken, 1859 | 2.63 - 3.03 | 1.00  (**±**1.00) | 3 | 0.54 | 0 | 0.21 |
| *Ophiocnida scabriuscula* (Lütken, 1859) | 4.45 | - | 1 | 0.18 | 0 | 0.07 |
| *Ophiophragmus brachyactis* H. L. Clark, 1915 | 4.45 | - | 1 | 0 | 0.12 | 0.07 |
| *Ophiostigma isocanthum* (Say, 1825) | 1.58 - 3.31 | 2.44  (**±**0.86) | 14 | 0.54 | 1.32 | 1.01 |
| *Ophiothrix (O.) angulata* (Say, 1825) | 0.63 - 4.79 | 10.38  (±5.79) | 156 | 9.89 | 12.24 | 11.31 |
| *Ophiactis quinqueradia* Ljungman, 1872 | 2.48 - 7.62 | 17.00  (**±**9.67) | 168 | 2.74 | 18.36 | 12.18 |
| *Ophiactis savignyi* (Müller & Troschel, 1842) | 1.3 – 1.5 | 1.4  (**±**0.14**)** | 2 | 0.36 | 0 | 0.14 |
| *Ophionereis reticulata* (Say, 1825) | 1.94 - 6.59 | 4.90 (**±**2.32) | 158 | 23.07 | 3.84 | 11.45 |
| *Ophionereis squamulosa* Koehler, 1914 | 2.46 - 5.33 | 3.89  (**±** 1.43) | 235 | 27.28 | 10.32 | 17.04 |
| *Ophionereis olivacea* H. L. Clark, 1900 | 3.15 - 3.75 | 3.00  (**±**1.58) | 5 | 0.18 | 0.48 | 0.36 |
| *Ophionereis dolabriformis* John & A. M. Clark, 1954 | 3.20 - 5.09 | 3.9  (**±**0.87) | 8 | 0.36 | 0.72 | 0.58 |
| *Ophiocoma echinata* (Lamarck, 1816) | 3.06 - 16.68 | 9.87  (**±**2.50) | 196 | 7.69 | 18.48 | 14.21 |
| *Ophiocoma wendtii* Müller & Troschel, 1842 | 2.71 - 15.07 | 9.00  (**±**5.04) | 28 | 0.73 | 2.88 | 2.03 |
| *Ophiopsila hartmeyeri* Koehler, 1913 | 1.30 - 6.90 | 4.10 (**±**1.93) | 75 | 5.31 | 5.52 | 5.43 |
| *Ophioderma appressa* (Say, 1825) | 4.18 - 7.89 | 5.02  (**±**2.47) | 166 | 10.07 | 13.32 | 12.03 |
| *Ophioderma cinerea* Müller & Troschel, 1842 | 4.96 - 9.67 | 5.51  (**±**1.69) | 126 | 7.14 | 10.44 | 9.13 |
| **Total of specimens** |  |  | **1379** | **546** | **833** | **100%** |

**TABLE 2.** Data on the size (measured by disk diameter), number of individuals and relative abundance of voucher specimens collected at Paraíba State continental shelf, Northeastern Brazil. SD = standard deviation.
